# Comparison of tWo hospital quality Improvement interventions on inappropriate measurement and SupplEmentation of vitamin D: the WISE-D study

**DOI:** 10.1186/s12877-026-07220-4

**Published:** 2026-02-21

**Authors:** Jerome K. Balsiger, Raphael Dettwiler, Blandine Mooser, Leonel Da Cunha Gonçalves, Marie Méan, Carole Elodie Aubert

**Affiliations:** 1 Department of General Internal Medicine, Inselspital, Bern University Hospital, University of Bern, Bern, Switzerland; 2 Department of prison medicine, Geneva University Hospital, University of Geneva, Geneva, Switzerland; 3 Division of Internal Medicine, Lausanne University Hospital, University of Lausanne, Lausanne, Switzerland; 4 Institute of Primary Health Care (BIHAM), University of Bern, Bern, Switzerland

**Keywords:** Vitamin D, Low-value care, Choosing Wisely, Quality improvement

## Abstract

**Background:**

Despite a lack of evidence, measurement and supplementation of vitamin D remain frequent in the general population, leading to significant and potentially avoidable healthcare costs. The aim of this study was to evaluate the impact of two quality improvement interventions (minimal vs. intensive) on inappropriate vitamin D measurement and supplementation in hospitalized patients and compare the two interventions.

**Methods:**

We conducted a pre-post intervention study on general internal medicine wards in Switzerland between 01 July 2016 and 31 December 2023. We compared a minimal intervention consisting of short guidelines in one hospital with a more intensive intervention including an e-learning, reminder e-mails and quizzes in a second hospital. Inappropriate measurement and supplementation of vitamin D were defined as measurement/supplementation in the absence of specific conditions associated with vitamin D deficiency (i.e., osteoporosis, osteomalacia, hepatic failure, malabsorption syndrome, chronic kidney disease with hyperparathyroidism, granuloma-forming disorders, or medications affecting vitamin D or bone metabolism). We used difference-in-differences with random-effects regression with bootstrap standard errors to estimate the overall intervention effect on inappropriate vitamin D measurement and supplementation across hospitals and compared the differences between hospitals.

**Results:**

Among 31,755 hospitalizations, the average monthly percentage of inappropriate vitamin D measurement and supplementation were 4.0% and 20.5%, respectively, over the entire study period. Compared to the pre-intervention period, there was a 1.3% reduction in inappropriate vitamin D measurements after the start of the interventions (*p* = 0.003), with no significant difference between interventions (*p* = 0.98). However, the rate of inappropriate vitamin D supplementation did not significantly change after intervention start (*p* = 0.81).

**Conclusion:**

Training interventions for residents were associated with a reduction in inappropriate vitamin D measurement, but not supplementation. The lack of significant difference between both interventions, even if not confirming equivalence, suggests that simpler, more passive strategies may be just as effective as more intensive approaches, offering a cost-efficient way to curb low-value care.

**Supplementary Information:**

The online version contains supplementary material available at 10.1186/s12877-026-07220-4.

## Introduction

Low vitamin D levels are common, reaching a prevalence of over 40% in the general population in Europe, and even 87% in older hospitalized adults [1, 2]. The increased prevalence in older adults is due to reduced skin synthesis, lower dietary intake, and impaired renal conversion of vitamin D to its active form [3, 4]. Vitamin D regulates calcium and phosphate metabolism and plays an important role in bone and tooth formation, while its role on other organ systems is unclear [5]. Low vitamin D levels have been associated with a higher risk of falls, fractures, autoimmune disorders, infections, cardiovascular disease, cancers, and type 2 diabetes [6]. However, vitamin D supplementation is effective only for a few conditions like osteoporosis, while there is no evidence that vitamin D supplementation in asymptomatic, community-dwelling populations has an effect on mortality or the incidence of fractures, falls, depression, diabetes, cardiovascular disease or cancer [7, 8]. Therefore, routine screening of individuals without a specific condition increasing the risk of vitamin D deficiency is not recommended [9, 10]. Despite the increased prevalence of vitamin D deficiency, guidelines also do not recommend systematic testing in older adults [9–11].

Nevertheless, measurement and supplementation of vitamin D remain frequent despite a lack of appropriate indication. This incurs significant potentially avoidable costs, evaluated as high as CHF 90 million yearly in Switzerland and USD 79 billion in the United States [12, 13]. To reduce this low-value practice, the Choosing Wisely initiative (including smarter medicine - Choosing Wisely Switzerland in the 2021 top-five list) and the US Preventive Services Task Force (in 2015) have published recommendations against systematic testing for or supplementation of vitamin D [2, 9, 10]. Furthermore, some limitations in reimbursement policies have been introduced, for example in Switzerland in July 2022 for measurement of vitamin D in a population without risk factor in ambulatory care settings [14].

However, while recommendations alone are typically insufficient to change behaviors [15, 16], the optimal intensity of intervention required to reduce low-value practices – such as inappropriate measurement and supplementation of vitamin D – remains unclear. The goal of this study was thus to evaluate the impact of two quality improvement interventions of different intensity (“minimal” versus “intensive”) targeting medical residents and chief residents (i.e., senior physicians in their last year of residency or who completed residency, and who closely supervise residents), on inappropriate vitamin D measurement and supplementation among older adults hospitalized on an acute medical ward.

## Methods

### Study design and setting

**Fig. 1:**
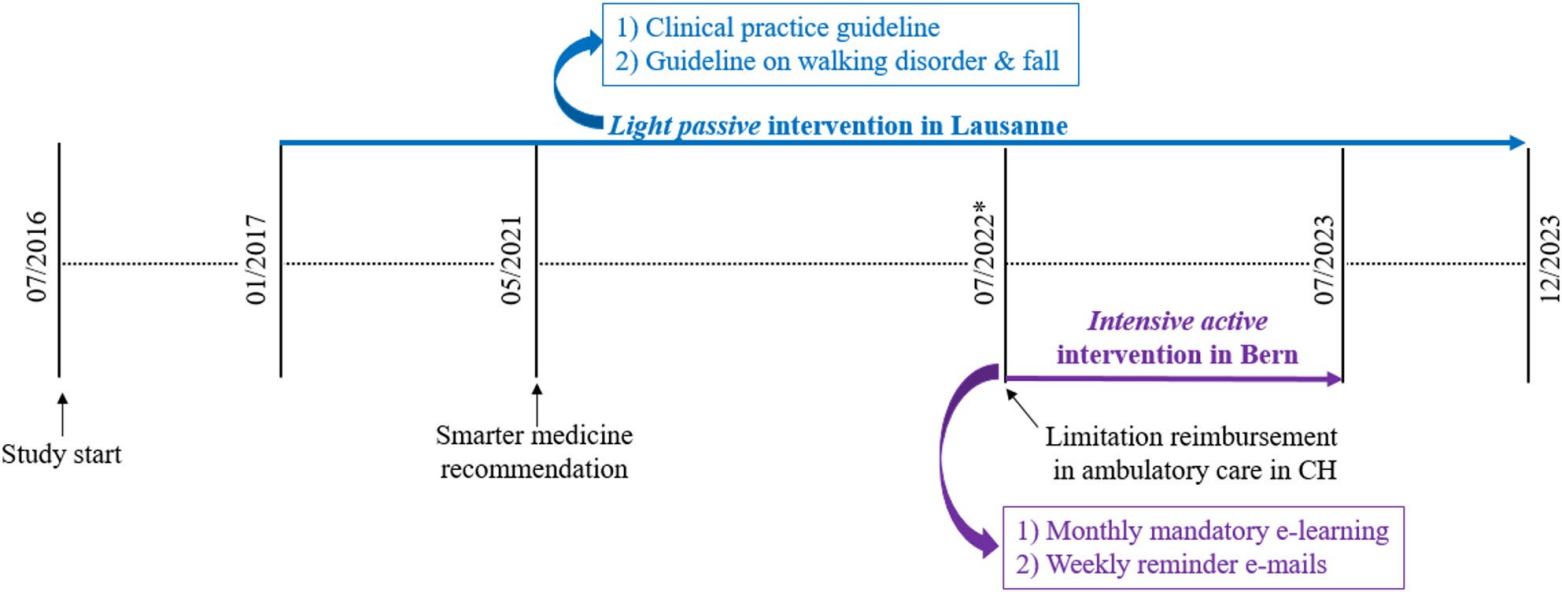
Study design and setting Figure legend: The study was conducted from July 2016 until December 2023. The figure displays the intervention periods in hospital 1 and hospital 2, as well as the time points of smarter medicine recommendation against generalized measurement of vitamin D, and the limitation of reimbursement in Switzerland for vitamin D measurement in ambulatory care

We conducted a pre-post intervention study at the departments of general internal medicine of two large teaching hospitals in Switzerland: Lausanne University Hospital (CHUV, French-speaking) and Bern University Hospital (Inselspital, German-speaking). The hospitals are similar in terms of patient population and offer training for residents in general internal medicine who are usually in their third to fifth year of residency. Chief residents represent about 20% of the medical team. Laboratory tests and medication are usually prescribed by the residents after discussion with the chief resident. Residents and chief residents assess the presence or absence of specific medical conditions or relevant medications through medical history and information from patient medical records. The study was conducted between 01 July 2016 and 31 December 2023 (Fig. 1).

### Study interventions and participants

We developed two interventions of different intensity, targeting all residents and chief residents of the participating departments (Table 1). The first intervention, implemented at hospital 1 and starting at month 7 (January 2017), was a minimal passive intervention consisting of internal guidelines about vitamin D measurement and supplementation, available for residents and chief residents. Two documents, developed by attending physicians, were included, (1) a one-sheet clinical practice guideline with a large title and very limited amount of text recommending to supplement vitamin D without measuring its blood level in specific situations (Supplement 1); and (2) a three-page guideline on walking disorder and falls in older adults (Supplement 2). The residents received the guidelines when they started their rotation in the department. A dashboard (digital interface) was implemented to monitor the frequency of vitamin D measurements. The data from the dashboard was used to provide feedback to residents (benchmarking), as well as during instructional sessions to support the performance of incoming residents.

**Table 1:** Description of study interventions

Aspect	Minimal Passive Intervention	Intensive Active Intervention
Setting	Hospital 1	Hospital 2
Implementation Period	01–2017-12/2023	07/2022-06/2023
Key Components	- One-sheet clinical practice guideline with clear title and minimal text that recommends supplementation without measurement. - Three-page document detailing evaluation, treatment, follow-up recommendations, and indications for vitamin D measurement and supplementation in older adults.	- Mandatory monthly e-learning with an initial comprehensive session with clinical vignettes and multiple-choice questions followed by short monthly quizzes. - Weekly reminder e-mails on appropriate indications for vitamin D measurement and supplementation.

The second intervention, implemented at hospital 2 from July 01 2022 until June 30 2023, was a more intensive and active intervention including a 15-minute interactive e-learning (Supplement 3), followed by short monthly quizzes (Supplement 4) and brief weekly reminder e-mails on the content of the e-learning (Supplement 5). The e-learning and quizzes were announced as compulsory, but there was no penalty for physicians who would not complete them. Completion was checked by an attending physician who sent personalized reminders to residents and chief residents if the training was not completed. At hospital 2, the residents were addressed when they started rotating in the department – either at the beginning of their employment, or upon returning to the medical ward after a rotation in another department. The chief residents were addressed at the beginning of the intervention period at hospital 2, or as soon as they newly started to work in the department.

### Study outcomes

We assessed two study outcomes: (1) inappropriate vitamin D measurement; and (2) inappropriate vitamin D supplementation over time. We defined inappropriate vitamin D measurement as testing conducted without the presence of specific medical conditions, including osteoporosis, osteomalacia, hepatic failure, malabsorption syndrome, chronic kidney disease with hyperparathyroidism, granuloma-forming disorders, or medications affecting vitamin D or bone metabolism. Similarly, inappropriate supplementation was defined as the use of vitamin D without any of those conditions. The outcomes were analyzed as the percentage of inappropriate vitamin D measurement and supplementation, respectively, among monthly hospitalizations within the two hospitals, from July 1 2016 to December 31 2023.

### Data collection and ethics

We studied data from patients aged 65 years and older who were admitted to the participating hospitals during the study period. The study was waived from ethical approval by the local Institutional Review Boards (“Kantonale Ethikkommission Bern” for Bern, and “Commission cantonale d’éthique de la recherché sur l’être humain CER-VD” for Lausanne), because it was considered as a quality improvement project and was thus not considered as human research according to Swiss regulation (project-ID 2022 − 01036). According to Swiss regulation, a specific consent is not required for data reuse. However, patients can explicitly refuse to have their data used for research, and such patients were therefore not included in the current project.

Patient data were abstracted from electronic health records and included age, sex, diagnoses, laboratory data (including vitamin D measurements and levels), and medication. We retrieved diagnoses using International Classification of Diseases, version 10, German Modification (ICD-10-GM) and calculated the Charlson Comorbidity Index using the version published by Quan et al. [17]. We used Anatomical Therapeutic Chemical codes (ATC codes) to extract medication data, including vitamin D supplementation.

### Data analysis

Descriptive statistics (means, ranges, and standard deviations) were used to describe hospitalizations and the percentages of inappropriate measurements and supplementations of vitamin D across the study periods and the two hospitals.

Linear regression analyses were conducted for each hospital separately to assess trends over time in the frequency of inappropriate measurement and supplementation, with outcomes modelled against consecutive month. Bootstrap standard errors with 1000 replications ensured robust estimates. The overall time effect over the study period was calculated by multiplying the monthly trend coefficient by 90 months. Differences in trends between hospitals were compared using a z-test.

To estimate the combined intervention effect across both hospitals, we used difference-in-differences with random-effects regression with bootstrap standard errors. This model included interaction terms for time, hospital, and intervention. We also compared differences between the minimal and the intensive interventions between hospitals.

In a sensitivity analysis, Auto Regressive Moving Average (ARMA) models with robust standard errors were applied separately to each hospital. Different models were tested, with the optimal one selected based on the lowest Bayesian Information Criterion. We included lagged variables (e.g., lag 1, denoted as “t–1”) to account for the influence of the outcome from the previous month(s) on the current value. In this context, “t” refers to the current month, while “t-1”, “t-2”, etc., indicate values from one or two months earlier, respectively. These lags are used to incorporate information from previous observations (autoregressive terms) or previous error terms (moving average terms) into the current prediction. A test for unknown structural breaks was conducted to assess the stability of regression coefficients over time. A z-test was then used to compare the results between hospitals.

Several diagnostic tests were conducted to verify the key assumptions underlying the regression models. These tests included checks for model specification, heteroscedasticity, normality of residuals, autocorrelation, eigenvalue stability, and multicollinearity. No major issues were found in any of these tests. Additionally, the dataset contained no missing values. Statistical significance was set at *p* < 0.05. All analyses were performed using Stata 15.0 (StataCorp).

## Results

### Study population

Over the 90-month study period, 13,689 hospitalizations were included at hospital 1 and 18,066 at hospital 2 (Table 2). Patient populations were largely similar between hospitals, with a mean patient age of 79.0 (SD 8.0) years and a mean Charlson Comorbidity Index of 6.6 (SD 3.0). The study period included an average of 156 (SD 37, range 74–246) monthly hospitalizations at hospital 1, and 201 (SD 23, range 133–254) at hospital 2. All residents (*N* = 101) and chief residents (*N* = 21) of hospital 2 completed the e-learning and the quizzes.

**Table 2:** Baseline characteristics

Characteristic	Hospital 1 (*N* = 13,689)	Hospital 2 (*N* = 18,066)
Age, mean (SD), years	78.9 (8.0)	79.0 (7.9)
Female, n(%)	7,609 (55.6)	9,680 (53.6)
Charlson Comorbidity Index, mean (SD)	6.6 (3.0)	6.7 (3.0)
Osteoporosis, n(%)	1,159 (11.5)	2,084 (11.5)
Hyperparathyroidism, n(%)	204 (1.5)	311 (1.7)
Chronic kidney disease, n(%)	3,307 (24.2)	4,681 (25.9)
Vitamin D supplementation, n(%)	5,990 (43.8)	7,732 (42.8)
Systemic corticosteroids, n(%)	3,676 (26.8)	3,550 (19.7)

Data are at hospitalization level

### Potentially inappropriate vitamin D measurements and supplementation over time

The mean monthly percentage of inappropriate vitamin D measurements was 3.6% (SD = 2.8%; range: 0.0–12.8%) at hospital 1, and 4.5% (SD = 4.0%; range: 0.0–18.3%) at hospital 2. The mean monthly percentage of inappropriate supplementation was 19.9% (SD = 4.0%; range: 8.1–30.3) at hospital 1, and 20.9% (SD = 3.4%; range: 14.2–29.1%) at hospital 2.

**Fig. 2:**
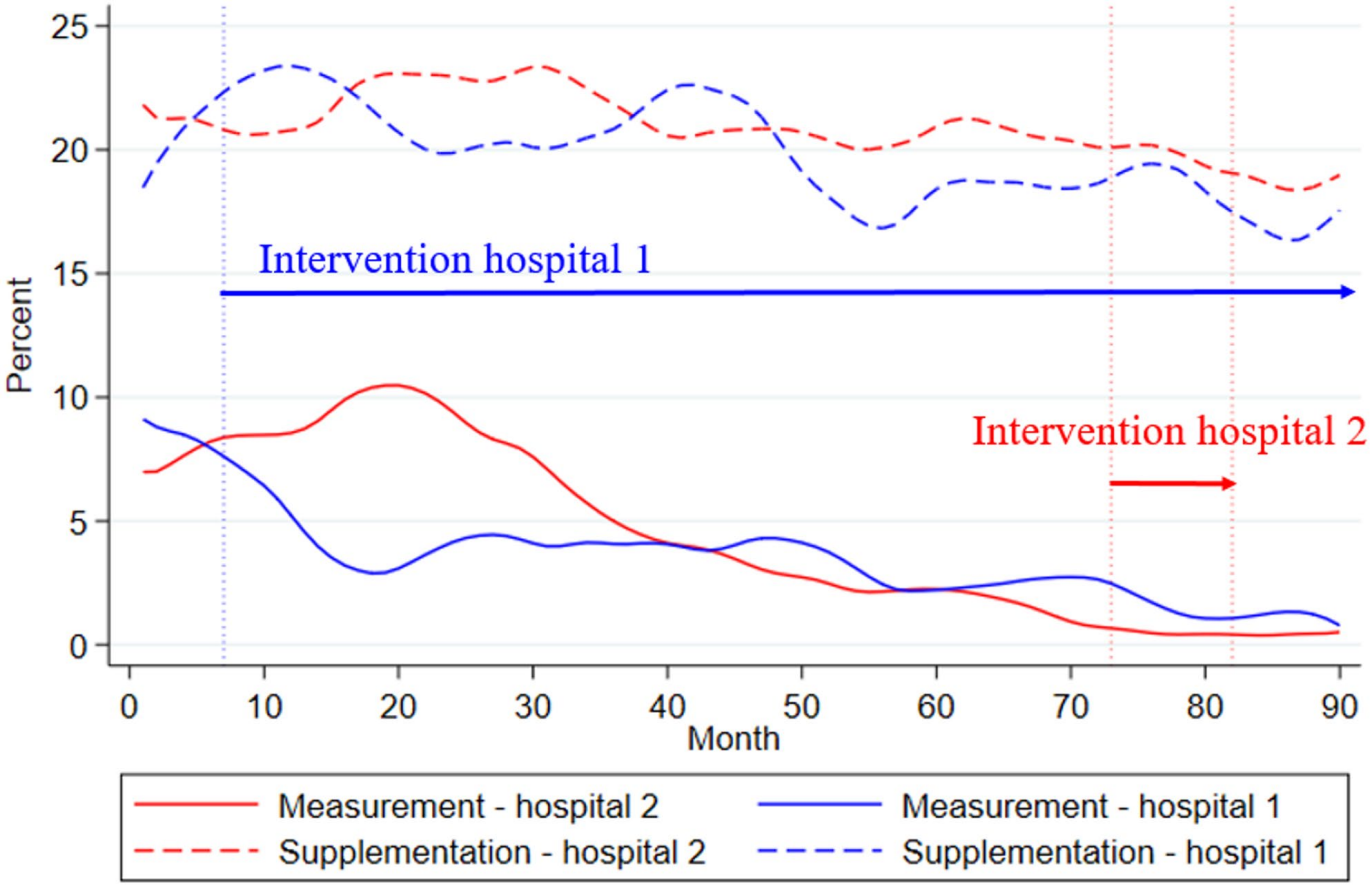
Percentages of potentially inappropriate measurements and supplementation of vitamin D over time across hospitals

There was a general reduction of inappropriate vitamin D measurement and supplementation in both hospitals over time (Fig. 2). Linear regression analyses confirmed significant reductions in inappropriate vitamin D measurements. However, the decrease of inappropriate measurements over the study period was higher in hospital 2 than in hospital 1 (11.2% vs. 5.6%, *p* < 0.001). There were no significant differences between hospitals regarding supplementation (5.4% vs. 3.2% respectively; *p* = 0.24).

### Main analysis

In the random-effects regression analyses, controlling for time, we found that, overall, inappropriate vitamin D measurements decreased by 1.3% (*p* = 0.005) across hospitals after the interventions started, without significant difference between hospitals (*p* = 0.98) and without interaction between hospitals and intervention periods (*p* = 1.00; Table 3).

On the other hand, there was a non-significant 0.3% decrease of inappropriate vitamin D supplementation across hospitals after the interventions started (*p* = 0.81; Table 3). There were also no significant differences between hospitals (*p* = 0.26) or interaction between hospitals and intervention periods (*p* = 0.54; Table 3).

**Table 3:** Estimated combined effect of the interventions

Variable	Measurement			Supplementation		
	b	95% CI	*p*-value	b	95% CI	*p*-value
Time (months)	−0.08	−0.10, −0.07	< 0.001	−0.05	−0.08, −0.03	< 0.001
Hospital	0.05	−3.46, 3.56	0.98	−2.70	−7.34, 1.95	0.26
Intervention	−1.26	−2.13, −0.38	0.005	0.26	−1.82, 2.33	0.81
Hospital*intervention	0.00	−3.45, 3.45	1.00	1.53	−3.40, 6.45	0.54
Constant	8.54	7.49, 9.60	< 0.001	23.28	21.98, 24.59	< 0.001

b= unstandardized regression coefficient from random effect linear regression with bootstrap standard errors

*Abbreviation*: *CI* Confidence interval

### Sensitivity analysis

At hospital 1, the best-fitting ARMA model revealed a non-statistically significant reduction in inappropriate vitamin D measurement after the minimal intervention started (4.1%, *p* = 0.07). A structural break was detected at month 20 (February 2018; *p* < 0.001). At hospital 2, the decrease was also non-statistically significant after the intensive intervention started (1.1%, *p* = 0.08). There was a structural break around month 22 (April 2018; *p* < 0.001). There were no significant differences between the two interventions (*p* = 0.20).

The minimal intervention was associated with a statistically non-significant reduction of 3.7% (*p* = 0.87) in vitamin D supplementation, and the intensive intervention with a 1.9% statistically non-significant reduction in vitamin D supplementation (*p* = 0.08). We did not identify any structural break in this analysis. The difference between the interventions was not significant (*p* = 0.36).

## Discussion

In this study, we evaluated two quality improvement interventions (minimal vs. intensive) to reduce inappropriate vitamin D measurement and supplementation and compared them with each other in two large teaching hospitals in Switzerland over a 90-month period. Both interventions were associated with a significant reduction of inappropriate vitamin D measurement, but not of supplementation. Interestingly, there was no statistical difference between the two interventions. Thus, regardless of the interventions, both hospitals exhibited a general decline in these low-value care practices over time.

While the reductions in inappropriate measurement and supplementation over time were modest compared to previous studies [18–20], when extrapolating to the number of inappropriate prescriptions over all hospitals, it could lead to significant cost savings across healthcare systems. Given the estimated potentially avoidable costs of potentially inappropriate vitamin D measurement [12, 13], the 1.3%-reduction in potentially inappropriate vitamin D measurement observed in our study could lead to cost savings as high as CHF 1.17 million in Switzerland, USD 5.2 million in Europe and USD 10.1 million in the United States.

Even if modest, this intervention could also help reduce potential side effects of vitamin D supplementation, which include nephrolithiasis, renal insufficiency, hypercalciuria, and hypercalcemia [21–25]. For example, a meta-analysis showed a 120% increased risk for hypercalcemia with doses over 3200–4000 IU/day compared to lower doses. Hypercalcemia is an adverse effect that is particularly worrying in older adults, given its association with altered mental status in this vulnerable population [26]. Furthermore, annual high-dose supplementation should be avoided, particularly in older adults, because it increases the risk of falls, for which age is already a well-known risk factor [27]. These potential harms of vitamin D supplementation should be balanced with potential benefits of supplementation in high-risk populations, as well as in older adults, particularly those living in institutions who could benefit from a supplementation of 700-1000IU/day to reduce the risk of falls [28].

The minimal intervention, which relied on internal guidelines, was associated with reductions in inappropriate vitamin D measurements comparable to those observed with the more intensive intervention. This suggests that passive strategies may be sufficient for certain low-value practices, such as inappropriate vitamin D measurement. Supporting this, a previous study found that an electronic health record clinical decision support tool significantly reduced vitamin D testing in a large safety-net system, with a 44% reduction in inpatient orders and 46% reduction in outpatient orders [18]. Similarly, another study showed that clinical decision support tools, which included screening guidelines and clinician alerts, significantly reduced inappropriate vitamin D screenings [19]. Simple interventions like educational memos and removal of tests from quick order screens in electronic health records were also successful in reducing unnecessary vitamin D testing in primary care [20].

However, such minimal, passive interventions cannot be generalized to all forms of low-value care, as other practices may require a more intensive approach – especially when external pressures are involved. One example is patient requests, which can lead physicians to prescribe low-value examinations, as discussing the necessity of the prescription is more time-consuming – especially under time pressure [29–31]. Furthermore, some physicians may seek to avoid conflicts with their patients. In such cases, a more intensive intervention or external incentives – such as reimbursement limitations, as implemented in Switzerland for vitamin D measurements in July 2022, may be more effective [14, 32].

Interestingly, while the interventions were not significantly associated with a reduction in inappropriate vitamin D supplementation, we observed a significant association between time and both potentially inappropriate vitamin D measurement and supplementation. While recommendations alone are usually not sufficient to change practices, it is possible that the accumulation of recommendations and guidelines published by multiple entities (such as Choosing Wisely, the US Preventive Task Force or scientific societies of multiple specialties), coupled to other initiatives (for example reimbursement limitations in some settings or clinical situations) are finally effective in reducing a low-value practice [8, 14, 15, 32].

To note, our study was conducted in teaching hospitals, where residents are closely supervised by chief residents and attending physicians, including researchers. Prescribing practice might be different if assessing board-certified physicians working in ambulatory care, who might have more time pressure and pressure from patients or be less updated on recent research. This might particularly influence prescriptions like vitamin D measurement and supplementation, given the ambiguous messages from research over the past decades [33]. Additional research should be conducted in other settings to assess whether results and trends in prescribing are similar.

### Clinical implications

The reductions in inappropriate vitamin D measurement and supplementation, while modest, carry significant clinical implications for healthcare systems. As discussed above, reducing unnecessary measurement and supplementation can lead to substantial cost savings by minimizing both the financial burden of excessive lab testing and the costs associated with inappropriate treatments. Further, it can help to reduce potential patient harm, such as the risk of hypercalcemia resulting from excessive vitamin D supplementation, as well as the risk of discomfort and complications from unnecessary blood draws. By streamlining these practices, physicians can not only enhance patient safety but also improve overall efficiency by conserving valuable healthcare resources – such as time, personnel, and equipment – that can be redirected toward higher-value care. In particular, the minimal intervention, with its lower resource demands, offers a compelling solution for divisions looking for cost-effective, scalable strategies to address overuse in clinical practice. It can be implemented with minimal disruption, while still driving meaningful improvements in care delivery, making it an attractive option for hospitals operating under resource constraints. Ultimately, the findings suggest that benefits for both healthcare providers and patients can be achieved with even modest changes in clinical practice.

### Strengths and limitations

This study is strengthened by a comprehensive and long-lasting dataset, with a substantial 90-month observation period that allows for a thorough analysis of trends over time. The study includes a high volume of hospitalizations, providing a robust sample size. Conducted in real-world clinical settings, this study reflects actual practices and challenges in hospital environments, adding to its external validity. Additionally, the inclusion of hospitals located in different regions with distinct cultural and linguistic backgrounds adds depth to the analysis, allowing the study to capture different perspectives and potentially different approaches to vitamin D measurement and supplementation. This diversity in setting and mentality provides a view across varied regions.

Despite the strengths of this study, several limitations must be acknowledged. First, the lack of randomization restricts the ability to draw firm causal conclusions about the effectiveness of the interventions. Second, the focus on university hospitals may limit the applicability of the results to smaller hospitals or other healthcare systems. Third, the reliance on ICD codes for diagnoses could lead to potential underreporting or misclassification of certain conditions, which may limit the comprehensiveness of the data. Fourth, some vitamin D measurement and supplementation might have been misclassified as inappropriate, given the sensitive definitions that we used. Fifth, a limitation of reimbursement for measurement of vitamin D in ambulatory care setting was introduced in July 2022 (at the same time than the intervention start in Bern) [14]. While this did not concern hospitalized patients, a pop-up in the electronic health records might have influenced physician decision to prescribe or not prescribe vitamin D measurement. Fifth, the pre-intervention period in hospital 1 was relatively short (seven month), which may have reduced precision in estimating baseline trends. Finally, the study lacked information on previous vitamin D measurements, which could have provided valuable context for assessing changes in clinical practice over time.

## Conclusion

Both a minimal and an intensive intervention were associated with reductions in inappropriate vitamin D measurement and supplementation over time, with no significant difference between them. The findings suggest that simpler, passive strategies may be just as effective as more intensive approaches, offering a cost-efficient way to curb low-value care. Future research should explore minimal yet effective interventions and the role of contextual factors in sustaining these improvements.

## Supplementary Information

Supplementary Material 1

Supplementary Material 2

Supplementary Material 3

Supplementary Material 4

Supplementary Material 5

## Data Availability

Data are available by the corresponding author upon reasonable request.

## Abbreviations

ATC: Anatomical Therapeutic Chemical
ICD-10-GM: International Classification of Diseases, version 10, German Modification
