# Supplementary material for: Comparison of tWo hospital quality Improvement interventions on inappropriate measurement and SupplEmentation of vitamin D: the WISE-D study
Source: BMC Geriatr. 2026 Feb 21;26:421. doi: 10.1186/s12877-026-07220-4 (PMC13032495; doi:10.1186/s12877-026-07220-4)
Supplement: Supplementary file 1 — Supplementary Material 1 [file 12877_2026_7220_MOESM1_ESM.pdf]

How does your patient mobilize?

clinical practice recommendations

Clinical practice recommendations
For a patient with gait disturbance, to be done on the first day of hospitalization:

- Determine the risk factors for falls
- Assess gait using the Tinetti test
- Check for a history of falls in the previous 12 months

Dare to use vitamin D without measuring it!

clinical practice recommendations

For patients with gait disorders:

• Vitamin D3 supplementation reduces the risk of falls at a daily dose of 800 IU/day

• Measuring 25-OH Vitamin D levels is only useful if there is a risk factor for deficiency (e.g., osteoporosis, malabsorption, obesity, etc.) or if a fracture occurs despite supplementation treatment
