# Supplementary material for: Comparison of tWo hospital quality Improvement interventions on inappropriate measurement and SupplEmentation of vitamin D: the WISE-D study
Source: BMC Geriatr. 2026 Feb 21;26:421. doi: 10.1186/s12877-026-07220-4 (PMC13032495; doi:10.1186/s12877-026-07220-4)
Supplement: Supplementary file 2 — Supplementary Material 2 [file 12877_2026_7220_MOESM2_ESM.pdf]

Gait disorders and falls in the older adults

1. Diagnostic approach

- **Medical evaluation** of transfers and gait upon admission (from day 1)
    - History (systematic if > 65 years): *Have you fallen in the last 12 months?* (If yes, frequency, mechanism, activity restrictions, fear of falling)
    - Gait evaluation by physicians:
        - POMA test (= Performance Oriented Mobility Assessment = Tinetti): 6 discriminating elements: difficulty rising from a chair (using upper limbs for assistance), instability in standing balance immediately, difficulty sitting down (falling into the chair), short stride, instability during turning, no foot clearance while walking
        - Risk-taking: hurrying to get up, not wearing shoes, not using assistive devices, relying on unstable supports, etc.
    - Neurological status (pallesthesia, Romberg test), musculoskeletal, visual or auditory disorders
- **Physical therapist evaluation**: Tinetti test, assistive devices, discharge orientation (e.g., home vs. rehabilitation)

2. Investigations

- **• Identification of risk factors:**
    - Intrinsic: malnutrition (Kondrup Nutritional Risk Screening), history of osteoporosis, use of assistive devices, lower limb or spine osteoarthritis, foot anomalies, lower limb sensory disorders, visual disorders, depressive syndrome (mini-GDS), cognitive disorders (Mini-COG, if positive MMSE or MoCA), acute confusion (CAM), urinary incontinence, orthostatic hypotension, acute illness, falls in the previous 12 months, multimorbidity, frailty, dependence, deconditioning, sarcopenia
    - Extrinsic: environment, polypharmacy (>4; STOPP/START criteria - brevimed 2nd edition), alcohol, medications (antipsychotics, antidepressants, hypnotics, benzodiazepines, diuretics, antiarrhythmics, opioids, anticholinergics, antihistamines, hypotensive agents, etc.)
- **Laboratory**: sodium, potassium, corrected calcium, creatinine, CK (if prolonged immobility)
    - **If polyneuropathy** and no recent investigations (<3 months): TSH, HbA1c, vitamin B12, immunoelectrophoresis. Second line, if clinical suspicion, consider syphilis and HIV screening
    - **No systematic vitamin D measurement**, except if:
        - Investigations of a calcium-phosphate imbalance or suspicion of hyperparathyroidism;
        - or if (in the absence of calcium-VitD3 substitution or recent vitamin D measurement) the patient presents: a) a symptomatology suggestive of osteomalacia/osteoporosis (muscle weakness, musculoskeletal pain); b) a long-term corticosteroid treatment; c) a spontaneous fracture suggesting osteoporosis
- **Orthostatism**: Schellong test (beware of postprandial orthostatic hypotension)

- **Dedicated imaging:** according to clinical signs (hematoma, pain, suspected fracture)
- **Fall:** Secure the patient, lift them from the ground, reassure the patient, and call the physician
    - **For any traumatic brain injury (TBI), assess the need for an emergency cervical CT and immobilization with a cervical collar**: if > 65 years old, dangerous mechanism (fall >1 meter or >5 stairs, axial impact on the vertex), extremity paresthesia, or active cervical rotation >45° is impossible
    - **For any TBI with coagulopathy**: INR > 1.2, platelets < 50 G/l, anticoagulation, anti-aggregation (except aspirin alone) -> **emergency cerebral CT**
    - Indications for cerebral CT and monitoring based on the severity of the TBI (see table)
    - **Note**: A fall without direct head trauma does not mean there is no risk of intracranial hemorrhage. In cases of significant brain atrophy, brain oscillation during the fall can strain perforating vessels, which may rupture.

Classification of head trauma

Classification	Diagnostic Criteria	Indications for Native Cerebral CT
Severe	GCS ≤8	Emergency cerebral CT + call resuscitation team
Moderate	GCS 9-12, persistent focal neurological deficit, skull fracture on clinical exam, penetrating cranial lesion	Emergency cerebral CT + intensive care evaluation
Minor	GCS 13-15, circumstantial anterograde or retrograde amnesia, new post-traumatic confusion or disorientation, brief loss of consciousness	Cerebral CT if:<br>• > 65 years<br>• Retrograde amnesia >30 min<br>• Fall >1 m height or >5 stairs<br>• GCS <15 at 2 hours post-trauma<br>• 1 episode of vomiting<br>• Post-traumatic seizures<br>• Suspected skull fracture (hemotympanum, raccoon eyes, CSF rhinorrhea/otorrhea, Battle's sign)
Simple	GCS 15, no amnesia, no confusion, no loss of consciousness	No routine CT required. Neurological monitoring every 2 hours for 12-24 hours (consciousness state, pupils, orientation).

3. Treatment

- **Correction/improvement of risk factors**
    - If antihypertensive treatment: review target systolic blood pressure. Reduce or stop antihypertensive treatment if orthostatic hypotension is documented; compression stockings (caution with arterial insufficiency)
    - If vasodilator treatment: nitrates especially in patch form, calcium antagonists, review indication
    - If benzodiazepine or hypnotic treatment: review indications and consider gradual withdrawal and substitution with less harmful alternatives (e.g., clomethiazole)
- If urinary urgency with or without incontinence: correct aggravating factors

- **Vitamin D supplementation**: no systematic initiation of supplementation, nor according to 25OH-vitamin D levels. Do not modify any ongoing supplementation
  - **Exceptions**: in the absence of hypercalcemia, in case of deficiency (especially in frail patients):
    - Severe: 25-OH-vitamin D <10 ng/ml (25 nmol/l): load 50,000 IU PO 1x/week for 6-8 weeks then 800 IU/day
    - Moderate: 25-OH-vitamin D >10 and < 20 ng/ml (>25 and < 50 nmol/l): load 50,000 IU PO 1x/week for 1-2 weeks then 800 IU/day
- **Calcium supplementation**: 500-1,000 mg/day depending on dietary intake (target 1,000-1,200 mg/day) in case of osteoporosis/osteomalacia. Favor diet with dairy products
- **Mobilization to the chair and/or walking 3x/day from day 1, with meals at the table 3x/day**
  - Prescribe physical therapy based on interprofessional assessment if mobility is altered: evaluation (Tinetti), static and dynamic postural balance work, muscle strengthening, choice/adaptation of assistive devices or orientation
- **Home adaptation**: installation of an emergency call system, visit from the home-based care, and occupational therapist.

4. driteria for discharge from acute ward

**Clinical status**	**No complicated fall in the last 24 hours**
Orientation	- Rehabilitation criteria: dependence in daily living activities, gait and balance disorder, organ involvement with functional impact or requiring stabilization of ongoing treatment, ability to do 2x20 minutes of physiotherapy per day<br>- Criteria for discharge home: Tinetti ≥15/28, proper use of assistive devices, transfers alone

5. Follow-up and discharge summary

Discharge summary	- Specify if gait disorder with/without fall, Tinetti<br>- Intrinsic and extrinsic factors, documentation of falls in the past 12 months<br>- Assistive devices and home adaptation proposals<br>- Modification of drug treatments according to STOPP/START criteria
