# Supplementary material for: Comparison of tWo hospital quality Improvement interventions on inappropriate measurement and SupplEmentation of vitamin D: the WISE-D study
Source: BMC Geriatr. 2026 Feb 21;26:421. doi: 10.1186/s12877-026-07220-4 (PMC13032495; doi:10.1186/s12877-026-07220-4)
Supplement: Supplementary file 3 — Supplementary Material 3 [file 12877_2026_7220_MOESM3_ESM.pdf]

WISE D – Reminder Emails

1.
TITLE: Vitamin D deficiency: always supplement?
E-MAIL CONTENT: NO! Only in situations that can be improved by supplementation: osteoporosis, hyperparathyroidism, long-term oral corticosteroid use (due to osteoporosis risk), osteomalacia, rickets, fractures suspected of osteoporosis..

2.
TITLE: Which foods are rich in vitamin D?
E-MAIL CONTENT:

Legumes?

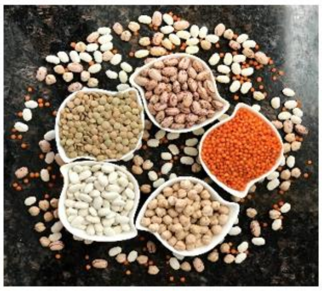

NO!

Diaries?

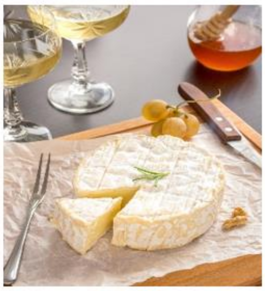

YES!

Fatty fish?

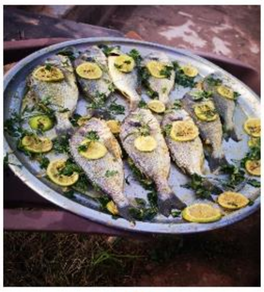

YES!

Red meat?

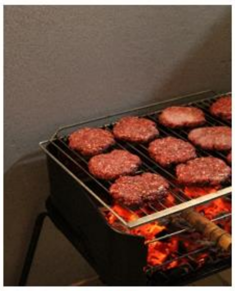

NO!

3.
TITLE: Should vitamin D deficiency and insufficiency be treated the same way?

E-MAIL CONTENT: NO! Vitamin D deficiency (<25 nmol/L): 6000 IU/d for 6 weeks, then 800-1000 IU/d. Vitamin D insufficiency (25-49 nmol/L): 800-1000 IU/d.
Link to the e-learning algorithm

4.
TITLE: Does vitamin D supplementation lead to hypercalcemia?

E-MAIL CONTENT: NO! There is no evidence that vitamin D supplementation at recommended doses leads to hypercalcemia.

5.
TITLE: Vitamin D activation?

E-MAIL CONTENT: Vitamin D (=Vitamin D_3) is inactive. It requires 2 hydroxylations to become active. The first hydroxylation occurs in the liver, producing 25-OH vitamin D_3. The second hydroxylation occurs in the kidney, producing 1,25-OH vitamin D_3.

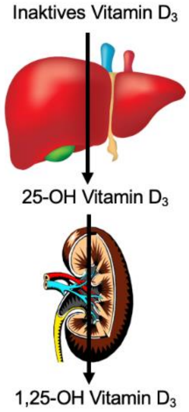

6.
TITLE: Vitamin D supplementation for everyone?
E-MAIL CONTENT: NO! There is no benefit to vitamin D supplementation in the general population.

7.
TITLE: Interpretation of vitamin D levels?

E-MAIL CONTENT:

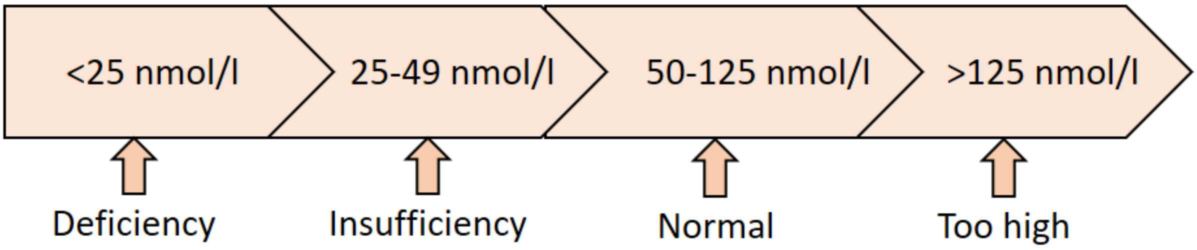

8.
TITLE: Should vitamin D be measured to investigate cognitive impairment?
E-MAIL CONTENT: NO! Because vitamin D supplementation has shown no benefit for the prevention or progression of cognitive impairment.

9.
TITLE: Should you measure 25-OH or 1,25-OH vitamin D?
E-MAIL CONTENT: 25-OH vitamin D (vitamin D storage).

10.
TITLE: Vitamin D supplementation for secondary hyperparathyroidism: which form?
E-MAIL CONTENT: First, supplementation with 25-OH vitamin D (cholecalciferol) should be done. If hyperparathyroidism persists despite adequate 25-OH vitamin D levels (50-125 nmol/L), continue with 1,25-OH vitamin D (calcitriol).

11.
TITLE: When do you measure vitamin D levels in the hospital?
E-MAIL CONTENT:

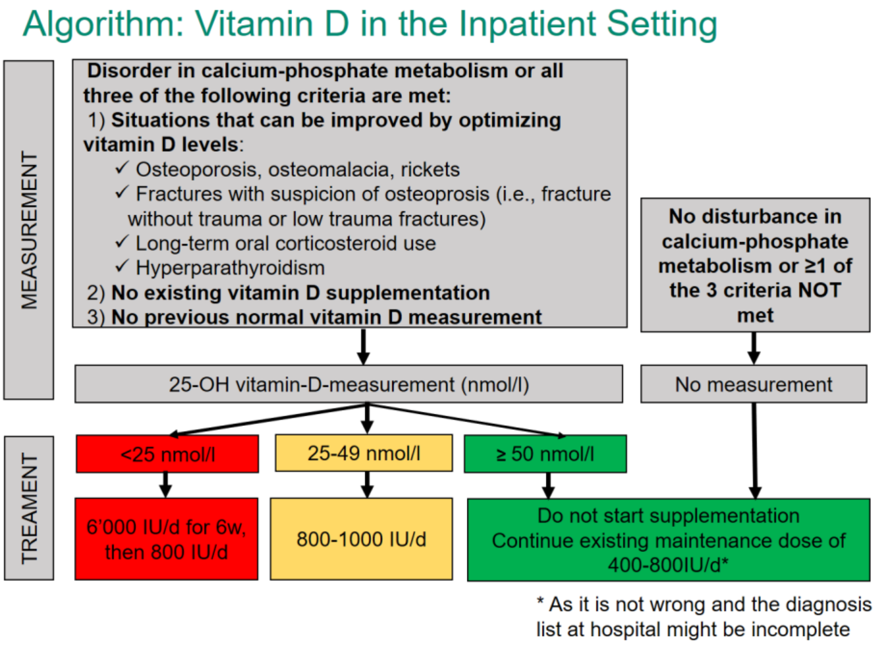

12.
TITLE: A patient is hospitalized due to a fall. Do you measure vitamin D?
E-MAIL CONTENT: NO! There is no evidence that vitamin D supplementation reduces the risk of falls in the general population. There is limited evidence for this in nursing home patients. Supplementation can be done without measurement.

13.
TITLE: When are 500 IU/drop vitamin D drops particularly useful?
E-MAIL CONTENT: During the first 6 weeks of treatment for vitamin D deficiency (6000 IU/d = 12 drops of 500 IU/drop).

14.
TITLE: Vitamin D-rich foods?
E-MAIL CONTENT: Eggs, salmon, cheese, mushrooms

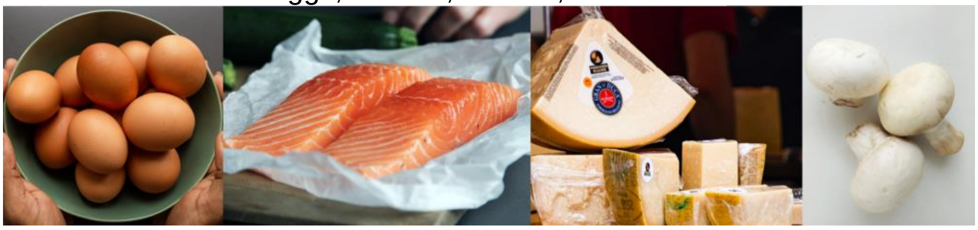

15.
TITLE: Is vitamin D supplementation useful for cancer prevention?
E-MAIL CONTENT: NO! There is no evidence for vitamin D supplementation for cancer prevention.

16.
TITLE: Should vitamin D always be measured in hospitalized patients with osteoporosis?
E-MAIL CONTENT: NO! If a vitamin D measurement has recently been performed and the level was normal, or if supplementation is already in place, no inpatient measurement is needed.

17.
TITLE: When should vitamin D levels be measured in the hospital?
E-MAIL CONTENT:

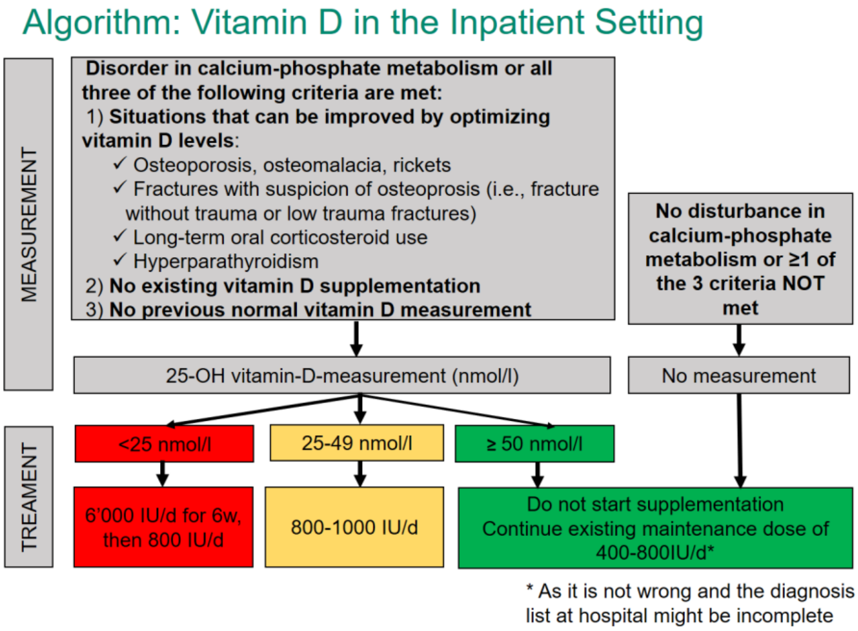

18.
TITLE: Should vitamin D supplementation be started in patients with infection?
E-MAIL CONTENT: NO! There is no evidence that vitamin D supplementation affects the incidence or course of infections.

19.
TITLE: Patient with osteoporosis, vitamin D 30 nmol/L 5 years ago, no therapy: Should vitamin D be measured?
E-MAIL CONTENT: YES! The 3 criteria are met.

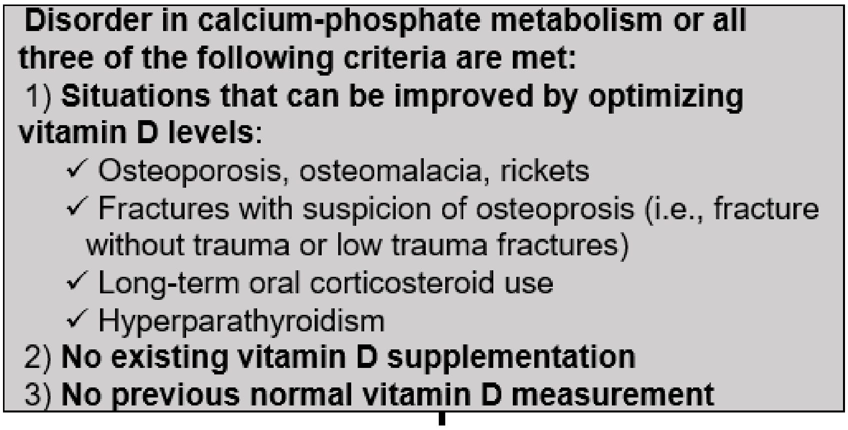

20.
TITLE: Vitamin D insufficiency (25-49 nmol/L): how to treat?
E-MAIL CONTENT:

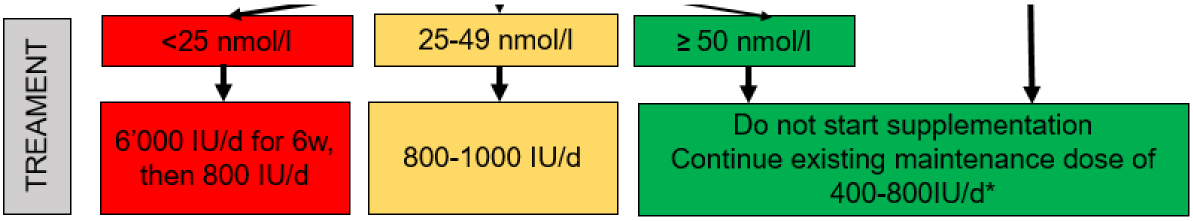

* As it is not wrong and the diagnosis list at hospital might be incomplete

21.
TITLE: Vitamin D supplementation for diabetes prevention?
E-MAIL CONTENT: NO! There is no evidence that vitamin D supplementation reduces the incidence of diabetes mellitus.

22.
TITLE: Vitamin D supplementation with inhaled corticosteroid use?
E-MAIL CONTENT: NO! Only with long-term oral corticosteroid use.

23.
TITLE: Main source of vitamin D?
E-MAIL CONTENT: 90% produced in the skin thanks to the ultraviolets produced by the sunlight.

24.
TITLE: Healthy patient, vitamin D 14 nmol/L: start supplementation?
E-MAIL CONTENT: NO! There is no benefit to vitamin D supplementation in the general population.

25.
TITLE: Vitamin D supplementation for the immune system?
E-MAIL CONTENT: NO! There is no evidence that vitamin D supplementation can strengthen the immune system and reduce the incidence of infections/cancer.

26.
TITLE: Vitamin D deficiency (<25 nmol/L): how to treat?
E-MAIL CONTENT:

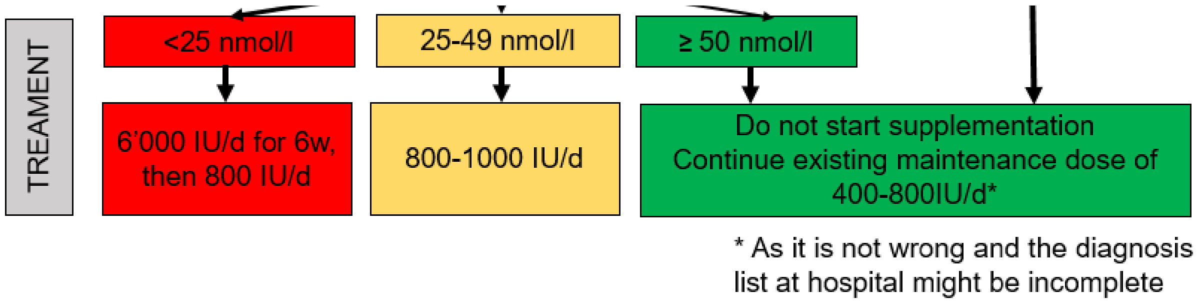

27.
TITLE: Does vitamin D supplementation cause nephrolithiasis?
E-MAIL CONTENT: NO! Not when vitamin D is supplemented at recommended doses.

28.
TITLE: Should vitamin D be supplemented for depression?
E-MAIL CONTENT: NO! There is no evidence that vitamin D supplementation improves depression.

29.
TITLE: Normal vitamin D level, healthy patient: start supplementation?
E-MAIL CONTENT: NO!

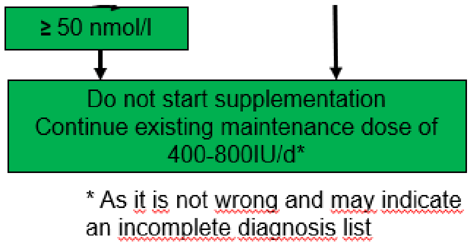

30.
TITLE: Vitamin D supplementation for the prevention of cardiovascular diseases?
E-MAIL CONTENT: NO! There is no evidence for vitamin D supplementation to prevent cardiovascular diseases.

31.
TITLE: Can vitamin D deficiency be corrected through diet alone?
E-MAIL CONTENT: NO! 10% of vitamin D comes from the diet. 90% is produced in the skin thanks to the ultraviolets of sunlight. A vitamin D-rich diet is usually not sufficient to correct vitamin D deficiency.

32.
TITLE: When should vitamin D be measured in the hospital?
E-MAIL CONTENT:

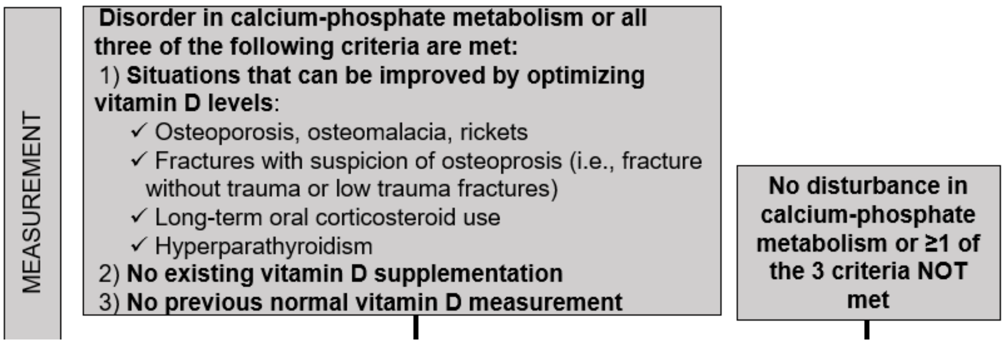

33.
TITLE: Vitamin D to reduce the incidence of fractures in the general population?

E-MAIL CONTENT: NO! The benefit of vitamin D supplementation in reducing the incidence of fractures exists only in high-risk populations (e.g., with osteoporosis).

34.
TITLE: Is a vitamin D level of 53 nmol/L high enough?
E-MAIL CONTENT: YES! 25-OH vitamin D levels between 50-125 nmol/L are high enough.

35.
TITLE: Vitamin D measurement and supplementation: when?
E-MAIL CONTENT:

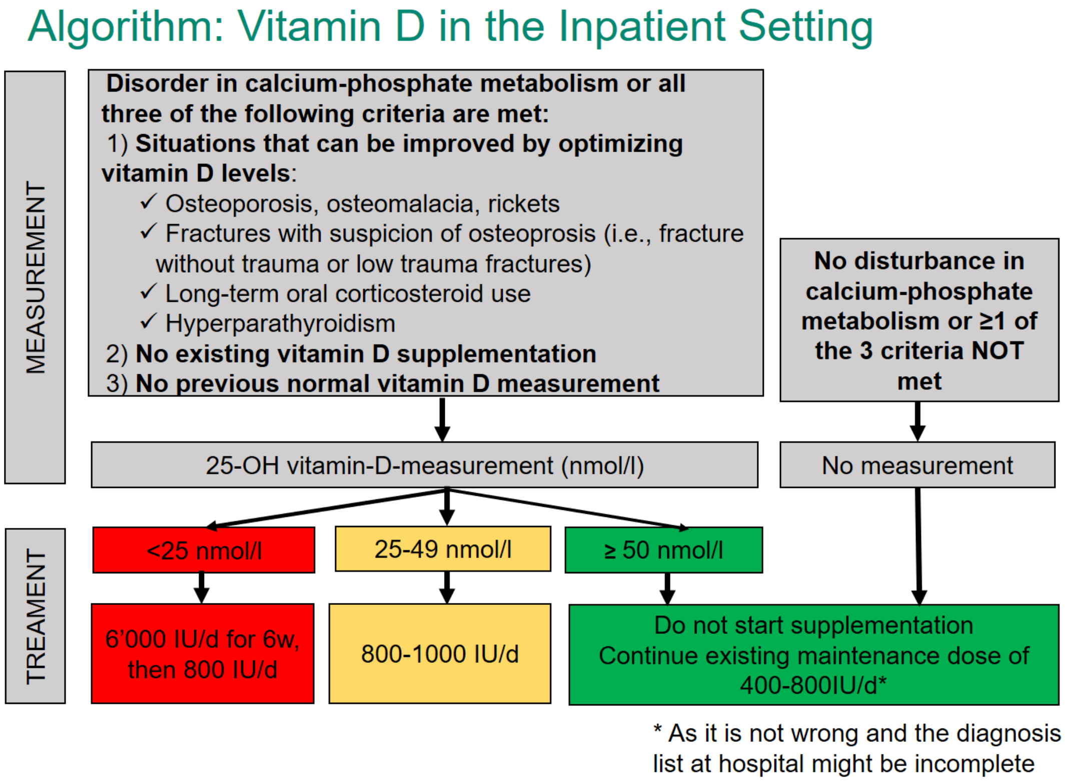

36.
TITLE: Vitamin D supplementation to reduce mortality?
E-MAIL CONTENT: NO! Low vitamin D levels have been associated with poor outcomes. However, there is no evidence that vitamin D supplementation reduces mortality.

37.
TITLE: BMI 17 kg/m²: Do you measure vitamin D?
E-MAIL CONTENT: NO! A low BMI by itself is not an indication for vitamin D measurement.

38.
TITLE: Hospitalized patient with vitamin D supplementation without a clear indication: stop?
E-MAIL CONTENT: NO!

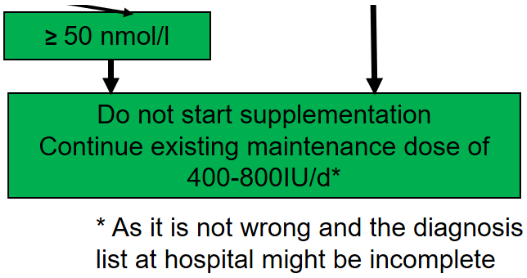

39.
TITLE: 500,000 IU of vitamin D as a single dose to correct a deficiency?
E-MAIL CONTENT: NO! High doses of vitamin D could lead to side effects.
