# Supplementary material for: Comparison of tWo hospital quality Improvement interventions on inappropriate measurement and SupplEmentation of vitamin D: the WISE-D study
Source: BMC Geriatr. 2026 Feb 21;26:421. doi: 10.1186/s12877-026-07220-4 (PMC13032495; doi:10.1186/s12877-026-07220-4)
Supplement: Supplementary file 4 — Supplementary Material 4 [file 12877_2026_7220_MOESM4_ESM.pdf]

VITAMIN D in the Inpatient Setting

Learning objectives

After completing this learning sequence, you will know

1) the indications for measuring vitamin D in the inpatient setting
2) the indications for vitamin D supplementation

Learning content

- What is Vitamin D
- Introduction to «smarter medicine»
- Vitamin D measurement in the inpatient setting
- Vitamin D **supplementation**

What is Vitamin-D?

Vitamin D is a fat-soluble hormone (referred to as a vitamin for historical reasons).
90% of vitamin D is produced in the skin with the help of the ultraviolet portion of sunlight.
A smaller amount (10%) comes from food: fatty fish (salmon, mackerel, etc.), eggs, mushrooms, dairy products.

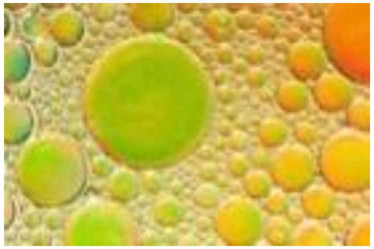

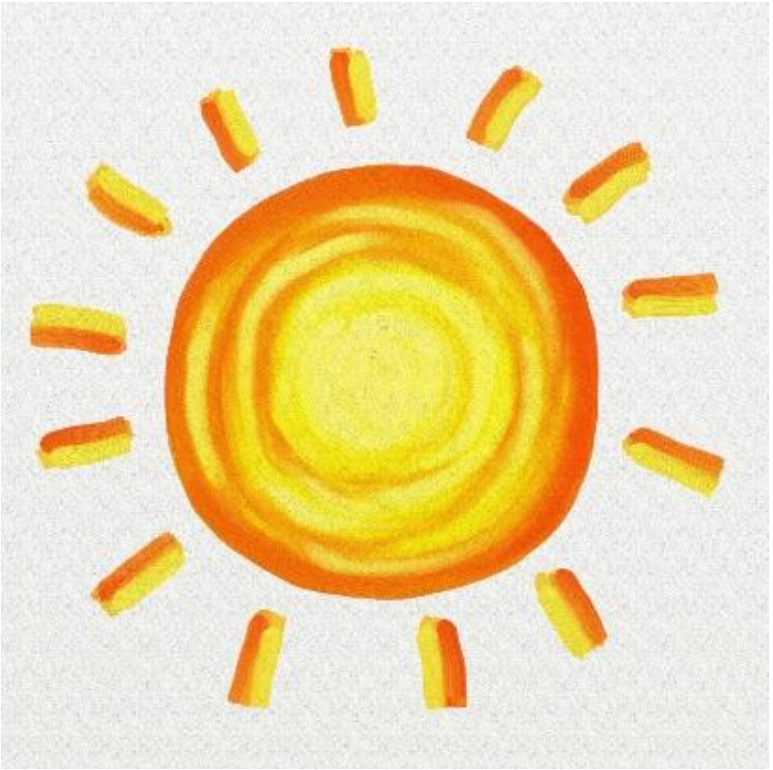

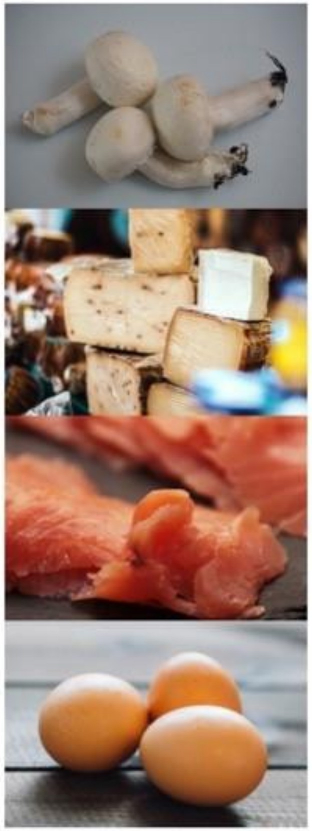

Activation of Vitamin D

Vitamin D (=Vitamin D_3) is inactive

It requires 2 hydroxylations to become active.

The 1st hydroxylation occurs in the liver, producing 25-OH vitamin D.

The 2nd hydroxylation occurs in the kidney, producing 1,25-OH vitamin D.

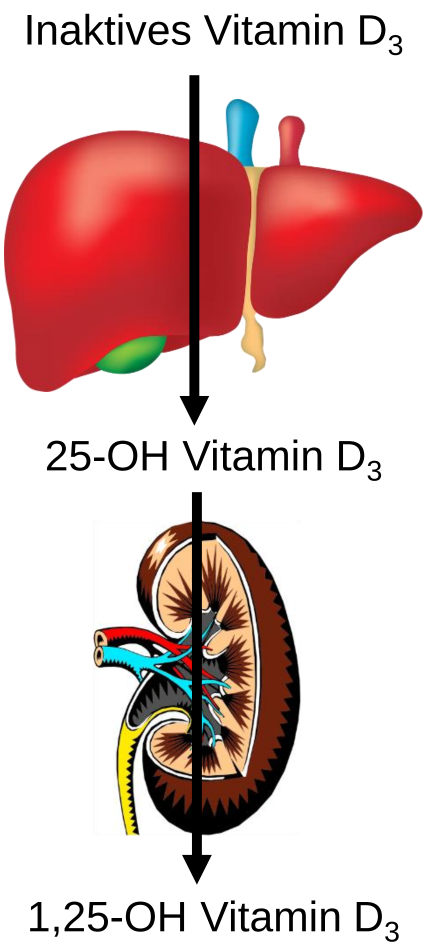

Vitamin-D-deficiency and -insufficiency

Vitamin D levels are categorized into 4 groups:

•Vitamin D **deficiency** is present when the 25-OH vitamin D level is <25 nmol/l.

•Vitamin D **insufficiency** is present when the 25-OH vitamin D level is 25-49 nmol/l.

•**Adequate** vitamin D supply is present when the 25-OH vitamin D level is 50-125 nmol/l.

•There is an increased risk of **side effects** from vitamin D when the 25-OH vitamin D level is ≥125 nmol/l.

« Smarter medicine – Choosing wisely »

The following principles should be adhered to when indicating investigations or therapies:

1) The benefit should be greater than any potential harm
2) An investigation should always lead to a consequence, such as a change in therapy. Additionally, an investigation should not lead to overtreatment.

These principles also apply to vitamin D measurement and supplementation.

A vitamin D measurement costs CHF 53.

smarter medicine

Choosing Wisely Switzerland

Vitamin D Measurement in the Inpatient Setting

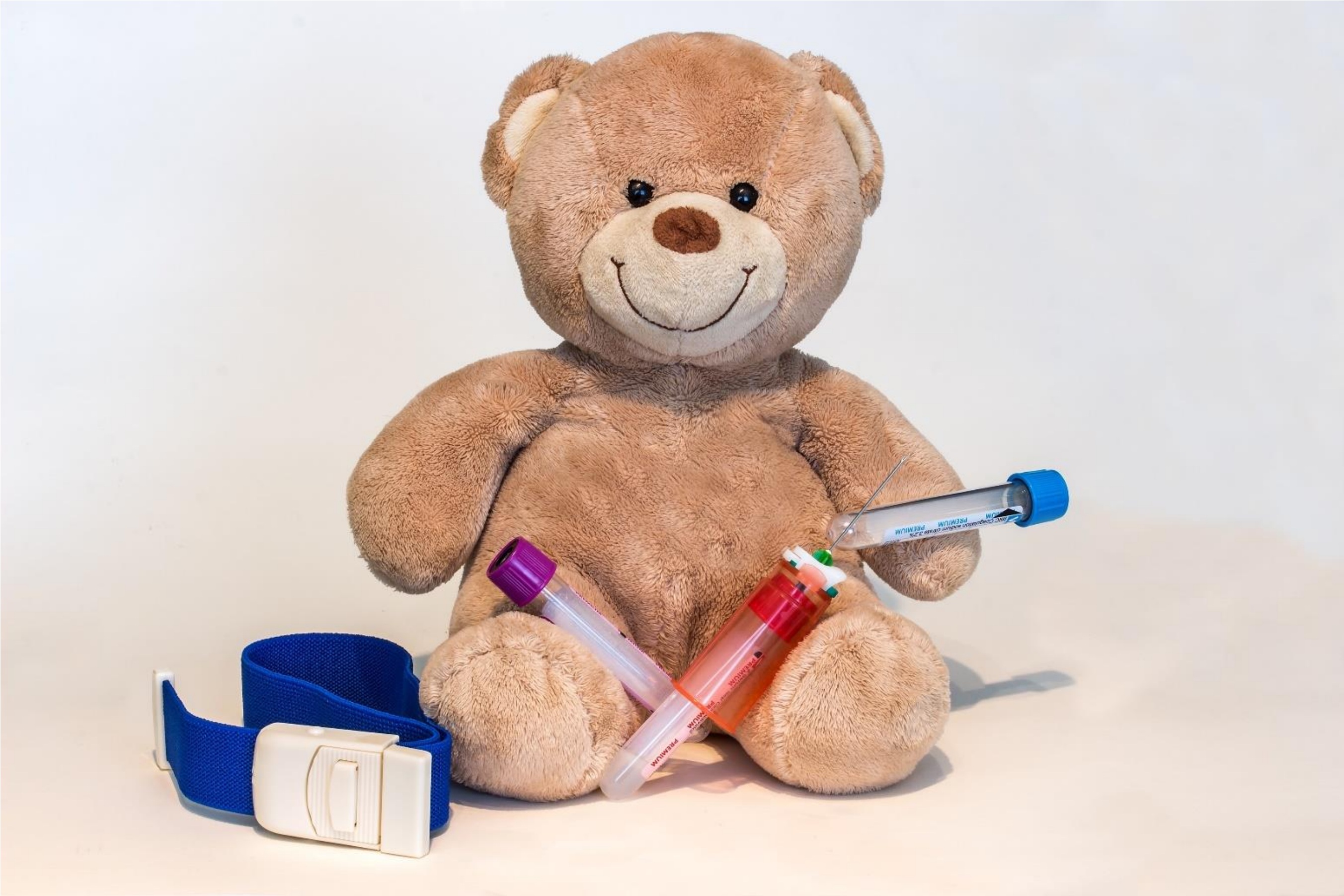

Question: Indications for Measurement

Before we list the indications for measuring vitamin D levels in the inpatient setting, let's see what you already know!

What are the indications for vitamin D measurement in the inpatient setting for patients without supplementation and without previous measurement?

1) BMI<18kg/m2
2) Osteoporosis
3) Fall
4) Sarcopenia
5) Cognitive impairment

Answer 2 is correct.
Only one answer is correct.
The only situations that indicate a need for vitamin D measure[illegible]
diseases, long-term oral corticosteroid use, and hyperparathy[illegible]

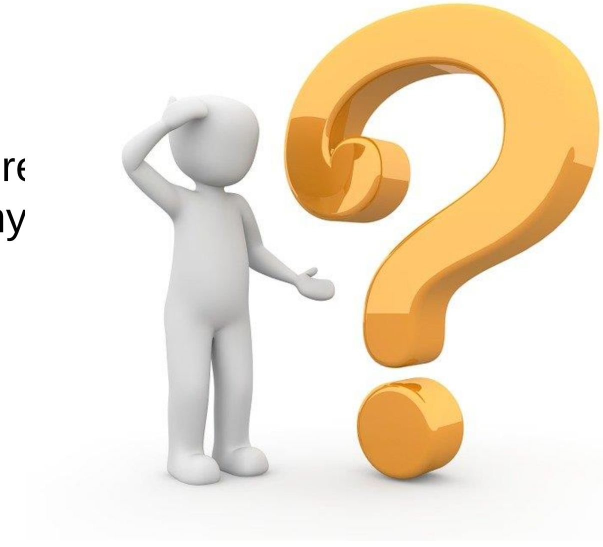

Indications for vitamin D measurement

In an inpatient setting, vitamin D should only be measured if **all three of the following criteria** are met:
:

1) A situation that can be improved by optimizing vitamin D levels.
2) No existing vitamin D supplementation.
3) No previous normal vitamin D measurement.

Situations that can be improved by optimizing vitamin D levels include:

- ✓ **Bone diseases:**
  - ✓ Osteoporosis
  - ✓ Osteomalacia
  - ✓ Rickets
  - ✓ Fractures suspected of osteoporosis = fractures without trauma or low-trauma fractures

- ✓ **Long-term oral corticosteroid use** (increased risk of osteoporosis)
- ✓ **Hyperparathyroidism**

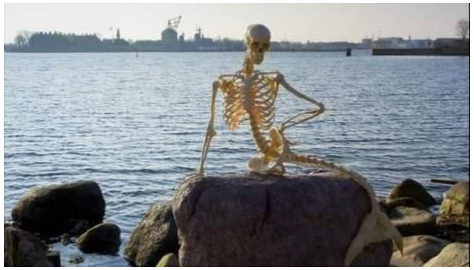

Which form of Vitamin D should be measured?

25-OH Vitamin D should be measured, even in cases of hyperparathyroidism of renal origin.

If the vitamin D level is <50 nmol/l in hyperparathyroidism of renal origin, supplementation with 25-OH vitamin D should be initiated.

If hyperparathyroidism persists despite an adequate 25-OH vitamin D level (50-125 nmol/l), the 1-OH hydroxylation process is not functioning properly.

In such cases, substitution with 1,25-OH vitamin D (Calcitriol) should be started without prior measurement of 1,25-OH vitamin D.

Question: measurement

Mrs. D. is hospitalized due to a fall from tripping without traumatic consequences. She lives alone at home, with support from home care services (Spitex).

Status: BMI 17.5 kg/m^2. MoCA 20 points. Otherwise, unremarkable status.

Do you measure the vitamin D?

1) Yes, because of the low BMI.
2) Yes, because of the fall..
3) Yes, because of the cognitive impairment.
4) Yes, for another reason
5) No.

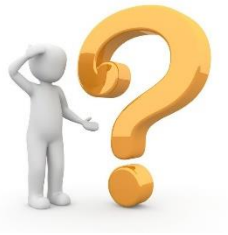

Answer: measurement

Correct Answer = 5).

Explanation of the answer:

Mrs. D. does not need a vitamin D measurement. She does not have a condition that can be improved by vitamin D supplementation. Therefore, there is no indication for a vitamin D measurement.

Cognitive impairment, low BMI, and a history of falls are not indications for vitamin D measurement.

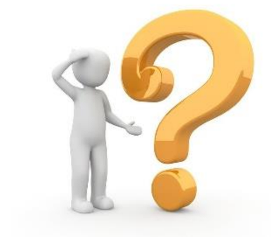

Vitamin-D-Supplementation

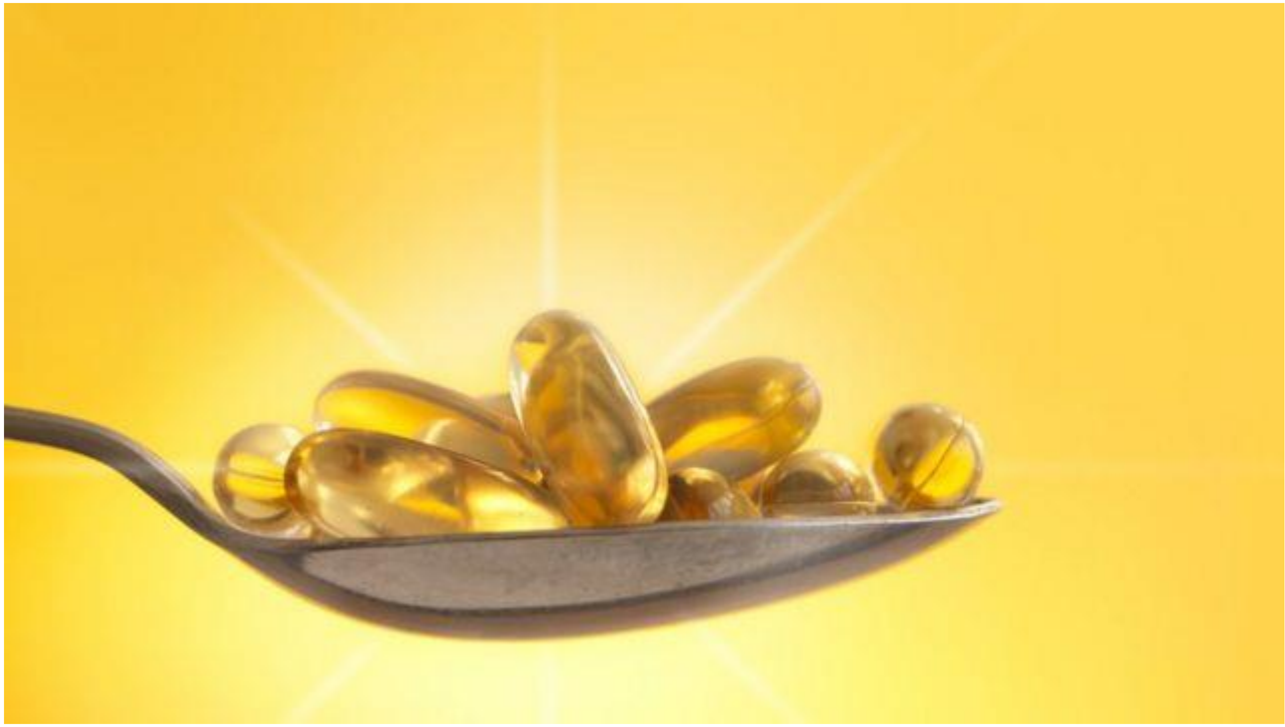

Question: Benefit of supplementation

Before we go through the evidence for the benefits and possible side effects of vitamin D supplementation, let's see what you already know!

When is vitamin D supplementation useful?

1) Fall prevention
2) Diabetes prevention
3) Dementia prevention
4) Osteoporosis therapy
5) Boosting the immune system

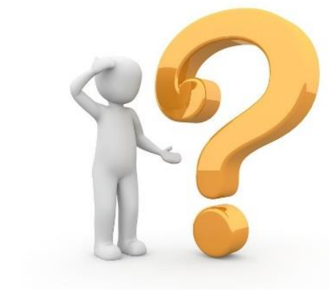

Answer: Benefit of supplementation

Correct Answer: 4)

Supplementation is indicated when the vitamin D level is low and there is a bone disease, long-term oral corticosteroid use, or hyperparathyroidism.

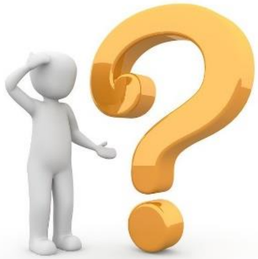

Benefit of supplementation

There is **no benefit of vitamin D supplementation** on the following outcomes in the general population:

- Mortality
- Physical performance
- Prevention of
  - Fractures
  - Diabetes mellitus
  - Cancer
  - Falls
  - Depression
  - Infections
  - Cardiovascular events

Side effects of supplementation

Vitamin D supplementation at standard doses does **not** lead to:

- Serious side effects
- Nephrolithiasis
- Hypercalcemia

Vitamin D intoxication only occurs with supplementation at excessively high doses. Patients present with symptoms of hypercalcemia.

Question: Vitamin-D-supplementation

Now let's see what you already know about vitamin D supplementation.

In a 60-year-old healthy patient, a vitamin D level of 30 nmol/l was measured. The patient had no indication for measurement, but now it has been done… What do you do?

1) No supplementation
2) Supplementation with 6000 IU daily for 6 weeks, dann 800 IU daily
3) Supplementation with 800 IU daily
4) Suppllementation with 1500 IU daily

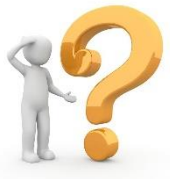

Answer: Vitamin-D-supplementation

Correct Answer = 1)

Explanation for answer 1: The patient has vitamin D insufficiency (25-50 nmol/l), but there is no situation that can be improved by supplementation. Therefore, she does not need supplementation.

Explanation for answer 2: This recommendation applies to vitamin D deficiency (<25 nmol/l) with a situation that can be improved by supplementation.

Explanation for answer 3: This recommendation applies to vitamin D insufficiency (25-50 nmol/l) with a situation that can be improved by supplementation.

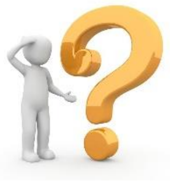

Indications for Supplementation

50% of the general population in Switzerland has a vitamin D deficiency *(Benhamou et al. SMW 2021).*

However, there is **no evidence of the benefit** of supplementation in the general population

Supplementation should only be undertaken **if both of the following criteria** are met :

1) 25-OH Vitamin-D-level <50 nmol/l
2) A situation that can be improved by supplementation:
    - ✓ Osteoporosis
    - ✓ Osteomalacia
    - ✓ Rickets
    - ✓ Fractures suspected of osteoporosis (fractures without trauma or low-trauma fractures)
    - ✓ Long-term oral corticosteroid use (osteoporosis prevention)
    - ✓ Hyperparathyroidism (primary (due to more severe symptoms when vitamin D is low) and secondary)

Supplementation Schemes

Supplementation with 100 IU of vitamin D leads to an increase in levels by 1.75-2.50 nmol/l.

There are two supplementation schemes, depending on vitamin D levels:

•For Vitamin D **deficiency** (<25 nmol/l):

- 100,000 IU every 2 weeks or 6000 IU/d orally for 6 weeks, followed by 800-1000 IU/d

•For Vitamin D i**nsufficiency** (25-50 nmol/l):

- 800-1000 IU/d

Question: Supplementation

In a 75-year-old patient with osteoporosis, a vitamin D level of 30 nmol/l was measured. What do you do?

1) No supplementation
2) Supplementation with 6000 IU daily for 6 weeks, then 800 IU daily
3) Supplementation with 800 IU daily

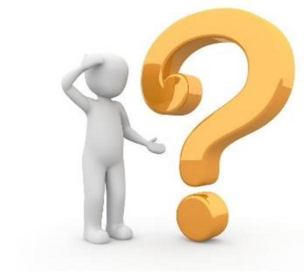

Answer: Supplementation

Correct = 3)

Explanation for answer 1: Supplementation is recommended because there is vitamin D insufficiency along with a bone disease.

Explanation for answer 2: This recommendation applies to vitamin D deficiency (vitamin D level <25 nmol/l).

Explanation for answer 3: For vitamin D insufficiency (vitamin D level 25-50 nmol/l) and an indication for supplementation (in this case, osteoporosis), supplementation with 800 IU daily is recommended.

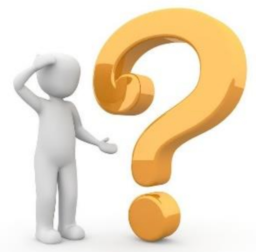

Vitamin-D medications

There are various medications that can be used depending on the supplementation dosage:

- Drops with 100 IU per drop
- Drops with 500 IU per drop
- Ampoules with 300,000 IU per ampoule (injection solution, but can also be administered orally)

The last two forms are particularly useful in cases of vitamin D deficiency.

Algorithm: Vitamin D in the Inpatient Setting

MEASUREMENT

Disorder in calcium-phosphate metabolism or all three of the following criteria are met:

1) **Situations that can be improved by optimizing vitamin D levels**:
   - ✓ Osteoporosis, osteomalacia, rickets
   - ✓ Fractures with suspicion of osteoprosis (i.e., fracture without trauma or low trauma fractures)
   - ✓ Long-term oral corticosteroid use
   - ✓ Hyperparathyroidism
2) **No existing vitamin D supplementation**
3) **No previous normal vitamin D measurement**

→ 25-OH vitamin-D-measurement (nmol/l)

No disturbance in calcium-phosphate metabolism or ≥1 of the 3 criteria NOT met

→ No measurement → Do not start supplementation

TREAMENT

25-OH vitamin D	Treatment
<25 nmol/l	6’000 IU/d for 6w, then 800 IU/d
25-49 nmol/l	800-1000 IU/d
≥ 50 nmol/l	Do not start supplementation<br>Continue existing maintenance dose of 400-800IU/d*

* As it is not wrong and the diagnosis list at hospital might be incomplete

Take home: Vitamin-D measurement

I) No systematic measurement of Vitamin D!

II) Measurement in the inpatient setting only in cases of calcium-phosphate metabolism disturbance or if all 3 criteria are met:

1) **A Situation that can be improved by optimizing vitamin D levels**:
   - ✓ Osteoporosis, Osteomalacia, Rickets
   - ✓ Fractures suspected of osteoporosis (fractures without trauma or low-trauma fractures)
   - ✓ Long term oral corticosteroid use
   - ✓ Hyperparathyreoidism
2) **No previous normal vitamin D measurement**
3) **No existing vitamin D supplementation**

III) Measure 25-OH (and not 1,25-OH) **vitamin D!**

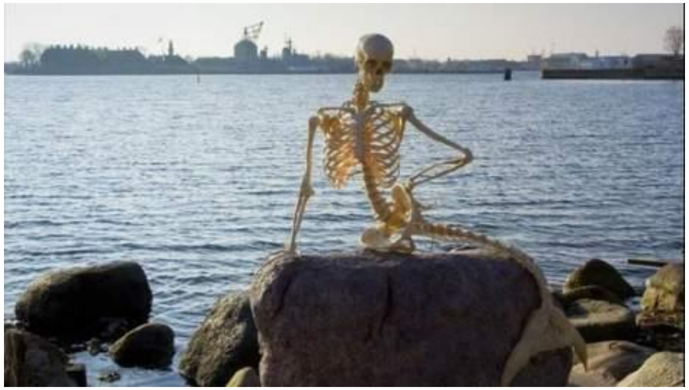

Take home: Vitamin-D-supplementation

I) Supplementation only if the following criterias are met:

1) 25-OH Vitamin D <50 nmol/l
2) A Situation that can be improved by optimizing vitamin D levels:

- ✓ Osteoporosis, Osteomalacia, Rickets
- ✓ Fractures suspected of osteoporosis (fractures without trauma or low-trauma fractures)
- ✓ Long term oral corticosteroid use
- ✓ Hyperparathyreoidism
- ✓Hypocalcemia

II) Higher dosis if initial level of 25-OH Vitamin D <25 nmol/l.

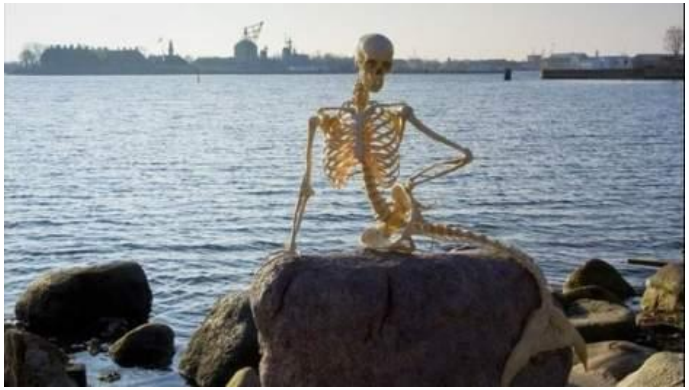

References

- Navarro et al. Vitamin D recommendations in clinical guidelines: A systematic review, quality evaluation and analysis of potential predictors. The International Journal of Clincial Practice. 2021.
- Kahwati et al. Screening for Vitamin D Deficiency in Adults Updated Evidence Report and Systematic Review for the US Preventive Services Task Force. JAMA. 2021.
- Holick et al.. Evaluation, Treatment, and Prevention of Vitamin D Deficiency: an Endocrine Society Clinical Practice Guideline. JCEM 2011.
- Bundesamt für Lebensmittelsicherheit und Veterinärwesen BLV. Empfehlungen zu Vitamin D. 2021.
- National Osteoporosis Society. Vitamin D and Bone Health: A Practical Clinical Guidelines for Patient Management.
- Haute Autorité de Santé. Utilité clinique du dosage de la vitamin D. Rapport d'évaluation. https://www.has-sante.fr/maintenance/index.html
