# Supplementary material for: Comparison of tWo hospital quality Improvement interventions on inappropriate measurement and SupplEmentation of vitamin D: the WISE-D study
Source: BMC Geriatr. 2026 Feb 21;26:421. doi: 10.1186/s12877-026-07220-4 (PMC13032495; doi:10.1186/s12877-026-07220-4)
Supplement: Supplementary file 5 — Supplementary Material 5 [file 12877_2026_7220_MOESM5_ESM.pdf]

Quiz 1

You are treating a 55-year-old patient who was admitted due to a distal radius fracture. She tripped over a branch while walking and fell from her own height. What do you do?

a. Protein electrophoresis

b. Measurement of vitamin D levels

c. Measurement of erythrocyte sedimentation rate (ESR)

d. No laboratory tests

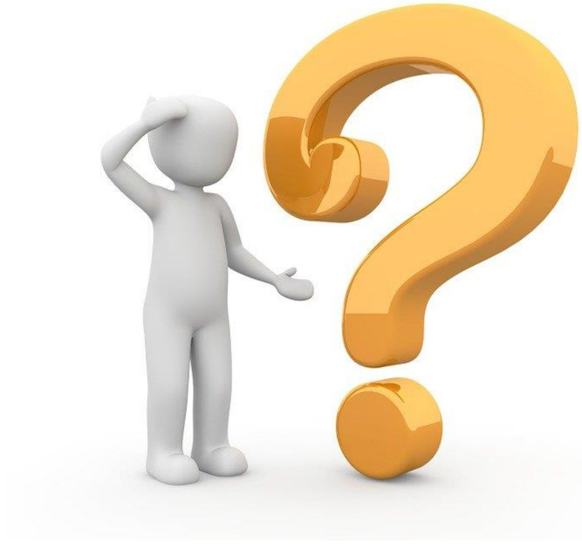

Answer 1

a. Protein electrophoresis would be indicated if multiple myeloma is suspected.

b. Correct

c. ESR measurement would be recommended if an inflammatory disease is suspected.

d. In case of suspected osteoporosis (fracture from a fall from one's own height), a laboratory test is indicated

Indications for vitamin D measurement

In the case of a fracture after a fall from one's own height, there is a suspicion of osteoporosis. Therefore, there is an indication to measure vitamin D levels. An evaluation of osteoporosis should also be conducted.

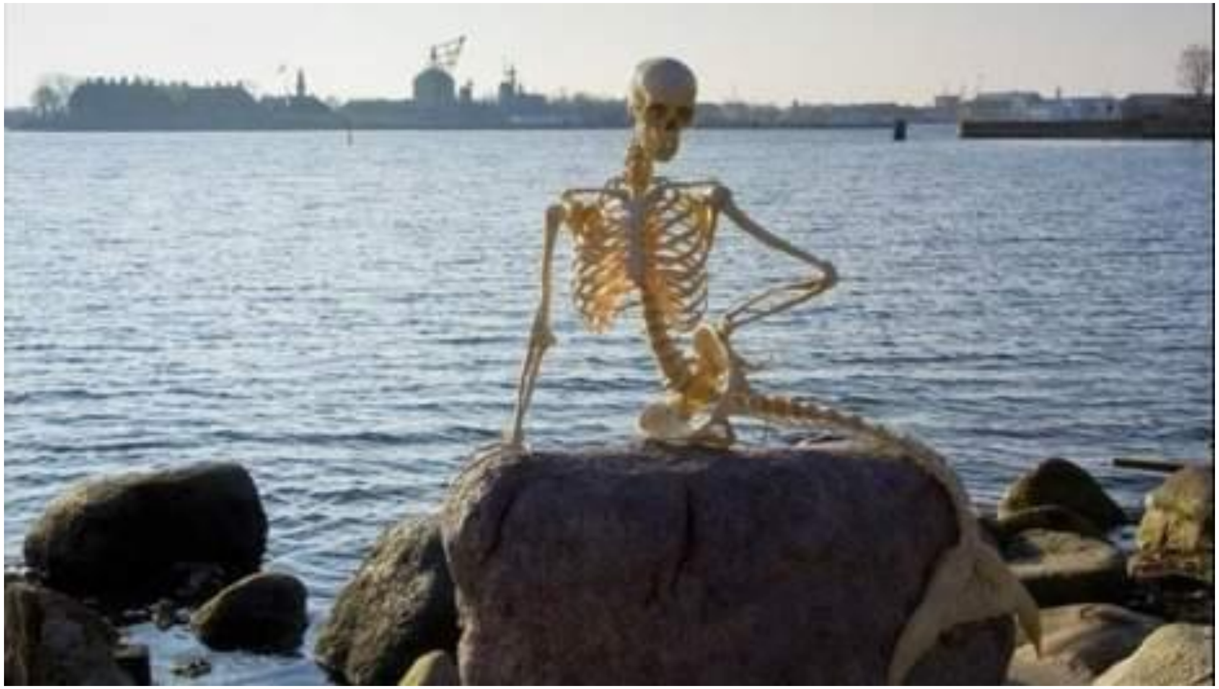

Indications for vitamin D measurement

In an inpatient setting, vitamin D should only be measured if **all three of the following criteria** are met:
:

1) A situation that can be improved by optimizing vitamin D levels.
2) No existing vitamin D supplementation.
3) No previous normal vitamin D measurement.

Situations that can be improved by optimizing vitamin d levels include:

- ✓ **Bone diseases:**
  - ✓ Osteoporosis
  - ✓ Osteomalacia
  - ✓ Rickets
  - ✓ Fractures suspected of osteoporosis = fractures without trauma or low-trauma fractures

- ✓ **Long-term oral corticosteroid use** (increased risk of osteoporosis)
- ✓ **Hyperparathyroidism**

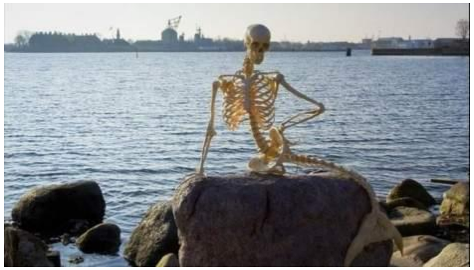

Algorithm: Vitamin D in the Inpatient Setting

MEASUREMENT

Disorder in calcium-phosphate metabolism or all three of the following criteria are met:

1) **Situations that can be improved by optimizing vitamin D levels**:
   - ✓ Osteoporosis, osteomalacia, rickets
   - ✓ Fractures with suspicion of osteoprosis (i.e., fracture without trauma or low trauma fractures)
   - ✓ Long-term oral corticosteroid use
   - ✓ Hyperparathyroidism
2) **No existing vitamin D supplementation**
3) **No previous normal vitamin D measurement**

→ 25-OH vitamin-D-measurement (nmol/l)

No disturbance in calcium-phosphate metabolism or ≥1 of the 3 criteria NOT met

→ No measurement

TREAMENT

<25 nmol/l → 6’000 IU/d for 6w, then 800 IU/d

25-49 nmol/l → 800-1000 IU/d

≥ 50 nmol/l → Do not start supplementation
Continue existing maintenance dose of 400-800IU/d*

No measurement → Do not start supplementation
Continue existing maintenance dose of 400-800IU/d*

* As it is not wrong and the diagnosis list at hospital might be incomplete

Quiz 2

You measure the vitamin D level in a patient with a fracture after a fall from their own height because you suspect osteoporosis. What exactly should you measure?

a. 1,25-OH vitamin D

b. 25-OH vitamin D

c. Total vitamin D

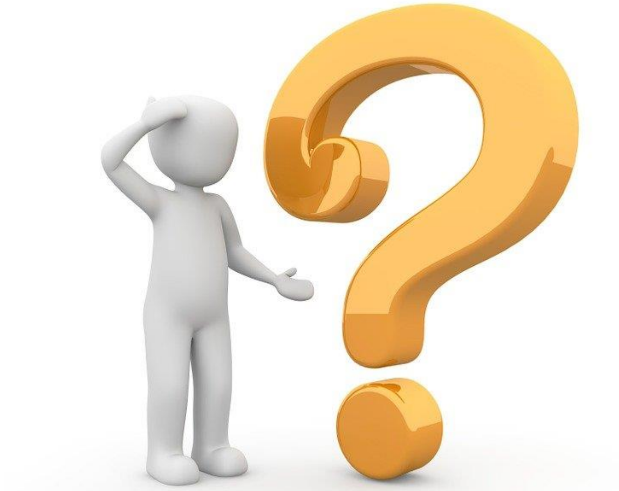

Answer 2

a. Wrong: 25-OH Vitamin D, which corresponds to the vitamin D storage, must be measured because it indicates the body's vitamin D reserves.

b. Correct

c. Wrong: 25-OH Vitamin D, which corresponds to the vitamin D storage, must be measured because it indicates the body's vitamin D reserves.

Activation and Storage of Vitamin D

The first hydroxylation of vitamin D occurs in the liver, producing 25-OH vitamin D. A second hydroxylation occurs in the kidney, producing 1,25-vitamin D. The 25-OH vitamin D, which corresponds to the vitamin D storage, must be measured.

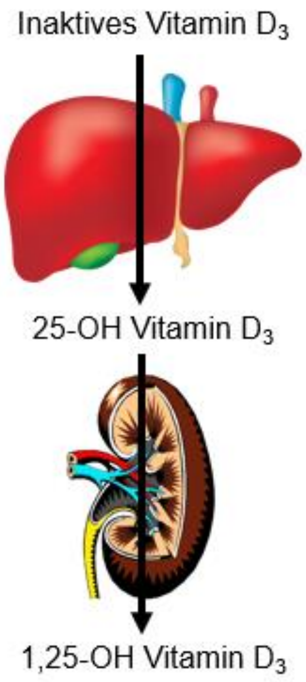

Which form of Vitamin D should be measured?

25-OH vitamin D should be measured, even in cases of hyperparathyroidism of renal origin.

If the vitamin D level is <50 nmol/l in hyperparathyroidism of renal origin, supplementation with 25-OH vitamin D should be initiated.

If hyperparathyroidism persists despite an adequate 25-OH vitamin D level (50-125 nmol/l), the 1-OH hydroxylation process is not functioning properly.

In such cases, substitution with 1,25-OH vitamin D (calcitriol) should be started without prior measurement of 1,25-OH vitamin D.

Algorithm: Vitamin D in the Inpatient Setting

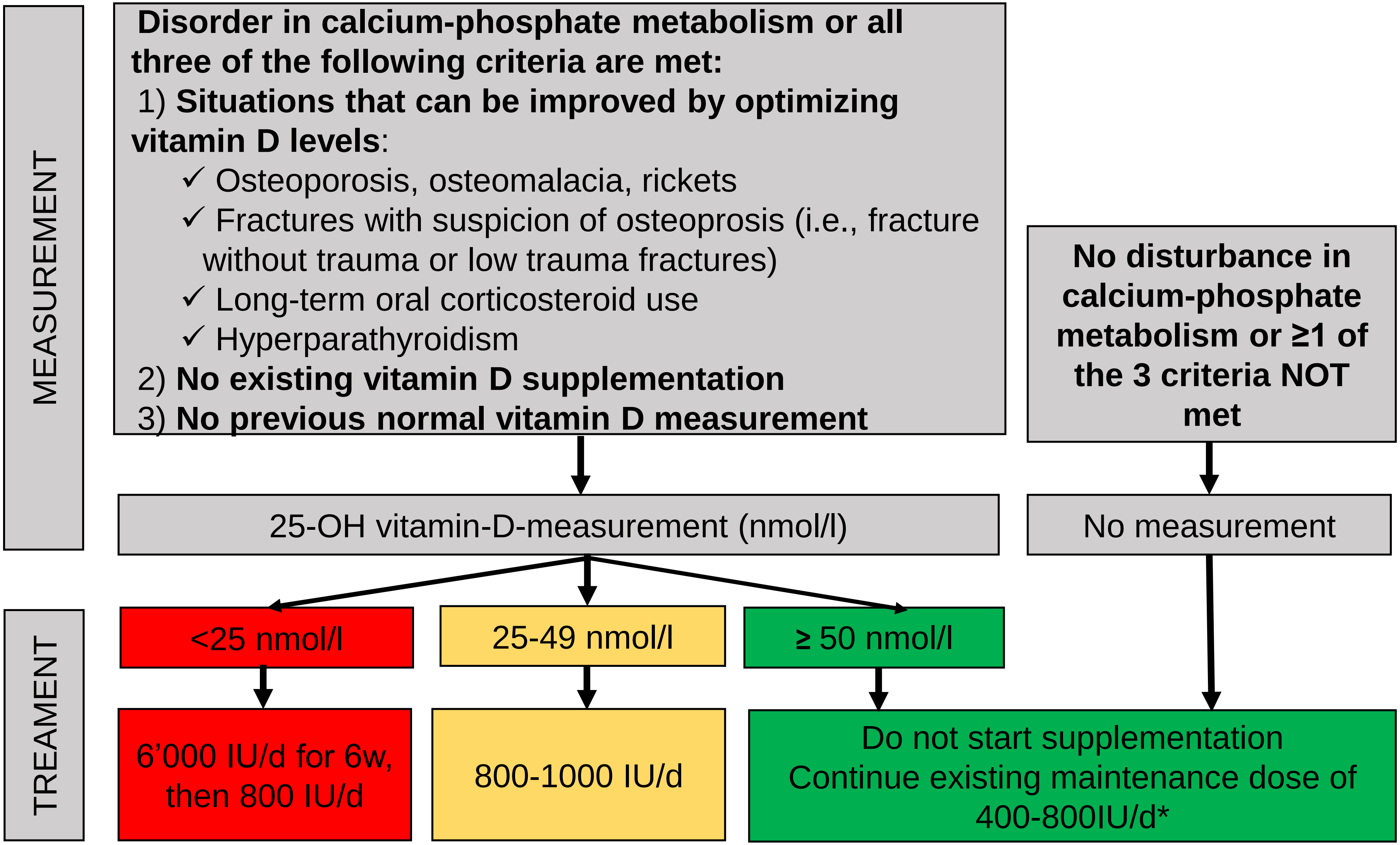

* As it is not wrong and the diagnosis list at hospital might be incomplete

Quiz 3

A patient with a fracture after a fall from her own height has a vitamin D level of 40 nmol/L. What is the diagnosis?

a. The vitamin D level is normal.

b. The vitamin D level is above normal.

c. There is a vitamin D deficiency.

d. There is a vitamin D insufficiency.

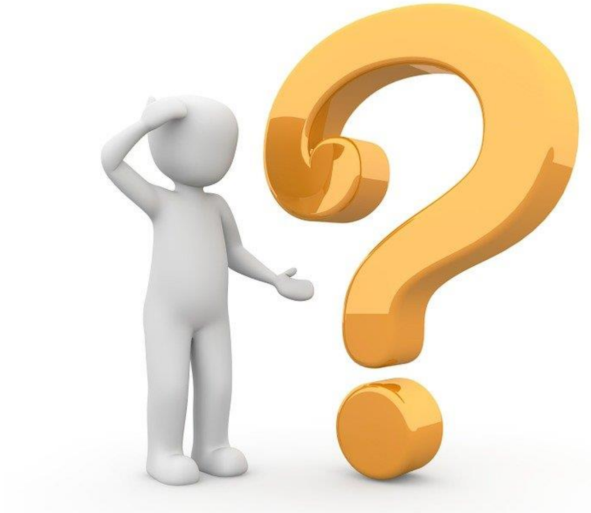

Answer 3

Correct answer is d

- Normal vitamin D level: 50-125 nmol/L
- Vitamin D insufficiency: 25-50 nmol/L
- Vitamin D deficiency: <25 nmol/L

Vitamin D deficiency and insufficiency

Vitamin D levels are categorized into 4 groups:

•Vitamin D **deficiency** is present when the 25-OH vitamin D level is <25 nmol/l.

•Vitamin D **insufficiency** is present when the 25-OH vitamin D level is 25-49 nmol/l.

•**Adequate** vitamin D supply is present when the 25-OH vitamin D level is 50-125 nmol/l.

•There is an increased risk of **side effects** from vitamin D when the 25-OH vitamin D level is ≥125 nmol/l.

Algorithm: Vitamin D in the Inpatient Setting

MEASUREMENT

Disorder in calcium-phosphate metabolism or all three of the following criteria are met:

1) **Situations that can be improved by optimizing vitamin D levels**:
   - ✓ Osteoporosis, osteomalacia, rickets
   - ✓ Fractures with suspicion of osteoprosis (i.e., fracture without trauma or low trauma fractures)
   - ✓ Long-term oral corticosteroid use
   - ✓ Hyperparathyroidism
2) **No existing vitamin D supplementation**
3) **No previous normal vitamin D measurement**

→ 25-OH vitamin-D-measurement (nmol/l)

No disturbance in calcium-phosphate metabolism or ≥1 of the 3 criteria NOT met

→ No measurement

TREAMENT

25-OH vitamin D	Treatment
<25 nmol/l	6’000 IU/d for 6w, then 800 IU/d
25-49 nmol/l	800-1000 IU/d
≥ 50 nmol/l	Do not start supplementation<br>Continue existing maintenance dose of 400-800IU/d*
No measurement	Do not start supplementation<br>Continue existing maintenance dose of 400-800IU/d*

* As it is not wrong and the diagnosis list at hospital might be incomplete

Quiz 4

How do you treat a patient with osteoporosis and a vitamin D level of 40 nmol/l?

a. No supplementation

b. 200 IU/d PO

c. 400 IU/d PO

d. 800-1000 IU/d PO

e. 6000 IU/d for 6 weeks, then 800-1000 IU/d PO

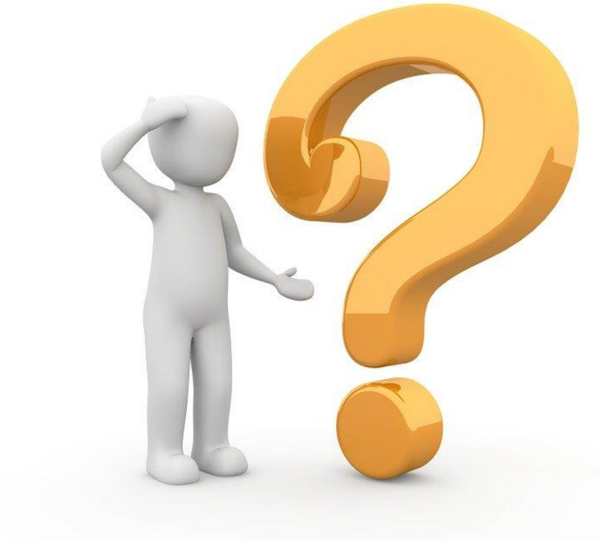

Answer 4

Correct answer: d

a: Supplementation is indicated in vitamin D insufficiency and osteoporosis.

b & c: These doses are too low for supplementing vitamin D insufficiency.

d: This is the recommended dose for vitamin D insufficiency (25-50 nmol/l).

e: This is the recommendation for vitamin D deficiency (<25 nmol/l).

Supplementation Schemes

Supplementation with 100 IU of vitamin D leads to an increase in levels by 1.75-2.50 nmol/l.

There are two supplementation schemes, depending on vitamin D levels:

•For Vitamin D **deficiency** (<25 nmol/l):

- 100,000 IU every 2 weeks or 6000 IU/d orally for 6 weeks, followed by 800-1000 IU/d

•For Vitamin D i**nsufficiency** (25-50 nmol/l):

- 800-1000 IU/d

Algorithm: Vitamin D in the Inpatient Setting

MEASUREMENT

Disorder in calcium-phosphate metabolism or all three of the following criteria are met:

1) **Situations that can be improved by optimizing vitamin D levels**:
   - ✓ Osteoporosis, osteomalacia, rickets
   - ✓ Fractures with suspicion of osteoprosis (i.e., fracture without trauma or low trauma fractures)
   - ✓ Long-term oral corticosteroid use
   - ✓ Hyperparathyroidism
2) **No existing vitamin D supplementation**
3) **No previous normal vitamin D measurement**

No disturbance in calcium-phosphate metabolism or ≥1 of the 3 criteria NOT met

25-OH vitamin-D-measurement (nmol/l)

No measurement

TREAMENT

<25 nmol/l	25-49 nmol/l	≥ 50 nmol/l
6’000 IU/d for 6w, then 800 IU/d	800-1000 IU/d	Do not start supplementation<br>Continue existing maintenance dose of 400-800IU/d*

* As it is not wrong and the diagnosis list at hospital might be incomplete

Question 5

You see a 63-year-old patient with inflammatory-type pain in the shoulder girdle and pelvis for two months. The status shows no local signs of inflammation in the joints. The ESR is elevated. The patient's vitamin D level has never been measured, and there is no vitamin D supplementation. You suspect polymyalgia rheumatica and begin treatment with prednisone. What else do you do?

a. Measure vitamin D levels: A deficiency could also explain the pain.

b. Measure vitamin D levels due to the initiated therapy.

c. Joint puncture.

d. Nothing further.

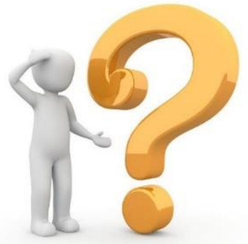

Answer 5

Correct answer: b

a. Vitamin D deficiency does not present with shoulder girdle pain or systemic inflammation. Vitamin D measurement is recommended with long-term corticosteroid therapy.

b. A measure of vitamin D is recommended for patients with long-term corticosteroid therapy.

c. Given the normal status, a joint puncture is not recommended.

d. A measure of vitamin D is recommended for patients with long-term corticosteroid therapy.

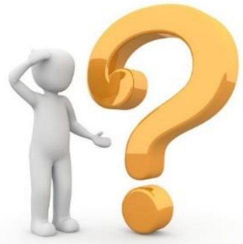

Indications for vitamin D measurement

In an inpatient setting, vitamin D should only be measured if **all three of the following criteria** are met:
:

1) A situation that can be improved by optimizing vitamin D levels.
2) No existing vitamin D supplementation.
3) No previous normal vitamin D measurement.

Situations that can be improved by optimizing vitamin D levels include:

- ✓ **Bone diseases:**
  - ✓ Osteoporosis
  - ✓ Osteomalacia
  - ✓ Rickets
  - ✓ Fractures suspected of osteoporosis = fractures without trauma or low-trauma fractures

- ✓ **Long-term oral corticosteroid use** (increased risk of osteoporosis)
- ✓ **Hyperparathyroidism**

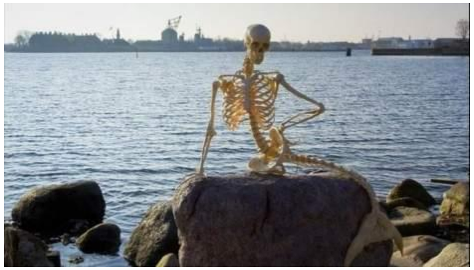

Algorithm: Vitamin D in the Inpatient Setting

MEASUREMENT

Disorder in calcium-phosphate metabolism or all three of the following criteria are met:

1) **Situations that can be improved by optimizing vitamin D levels**:
   - ✓ Osteoporosis, osteomalacia, rickets
   - ✓ Fractures with suspicion of osteoprosis (i.e., fracture without trauma or low trauma fractures)
   - ✓ Long-term oral corticosteroid use
   - ✓ Hyperparathyroidism
2) **No existing vitamin D supplementation**
3) **No previous normal vitamin D measurement**

→ 25-OH vitamin-D-measurement (nmol/l)

No disturbance in calcium-phosphate metabolism or ≥1 of the 3 criteria NOT met

→ No measurement

TREAMENT

25-OH vitamin D result	Treatment
<25 nmol/l	6’000 IU/d for 6w, then 800 IU/d
25-49 nmol/l	800-1000 IU/d
≥ 50 nmol/l	Do not start supplementation<br>Continue existing maintenance dose of 400-800IU/d*
No measurement	Do not start supplementation<br>Continue existing maintenance dose of 400-800IU/d*

* As it is not wrong and the diagnosis list at hospital might be incomplete

Quiz 6

You measure the vitamin D level in a patient under long-term corticosteroid therapy. The 25-OH vitamin D level is 60 nmol/L. What do you do?

a. Supplement with 800 IU/d.

b. Supplement with 400 IU/d.

c. No supplementation.

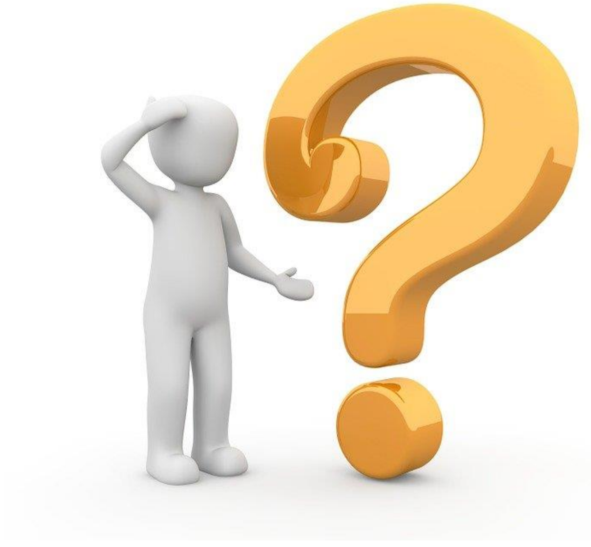

Answer 6

Correct answer: c

No supplementation is needed if vitamin D levels are normal, even with long-term corticosteroid therapy.

Indications for Supplementation

50% of the general population in Switzerland has a vitamin D deficiency *(Benhamou et al. SMW 2021).*

However, there is **no evidence of the benefit** of supplementation in the general population

Supplementation should only be undertaken **if both of the following criteria** are met :

1) 25-OH vitamin-D level <50 nmol/l
2) A situation that can be improved by supplementation:
   - ✓ Osteoporosis
   - ✓ Osteomalacia
   - ✓ Rickets
   - ✓ Fractures suspected of osteoporosis (fractures without trauma or low-trauma fractures)
   - ✓ Long-term oral corticosteroid use (osteoporosis prevention)
   - ✓ Hyperparathyroidism (primary (due to more severe symptoms when vitamin D is low) and secondary)

Algorithm: Vitamin D in the Inpatient Setting

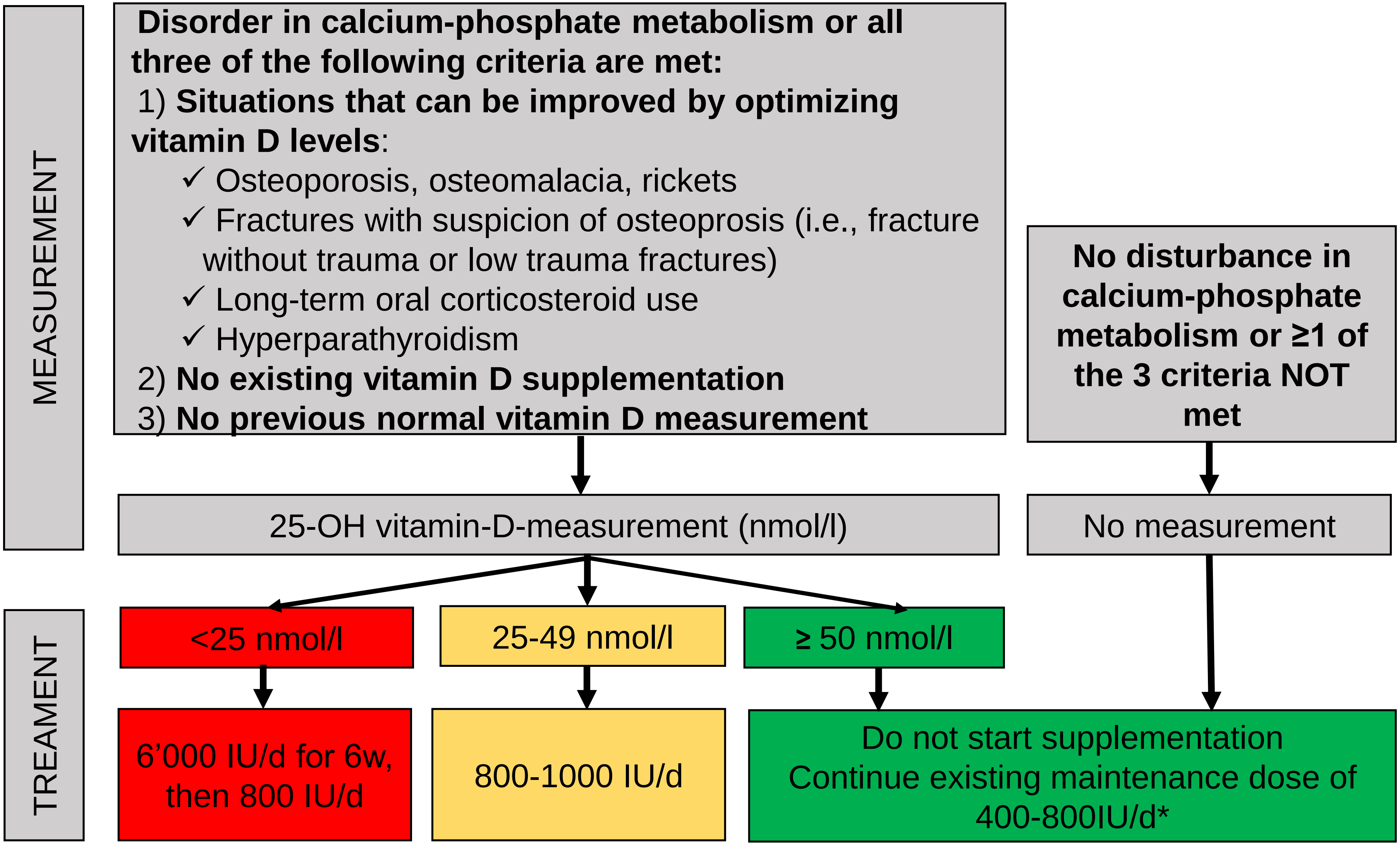

* As it is not wrong and the diagnosis list at hospital might be incomplete

Quiz 7

A patient with vitamin D deficiency asks how they can naturally increase their vitamin D levels. What do you suggest?

a. Go in the sun daily for 15 minutes without sunscreen.

b. Eat lean fish (e.g., sole).

c. Eat fatty fish (e.g., salmon, mackerel).

d. Consume dairy products.

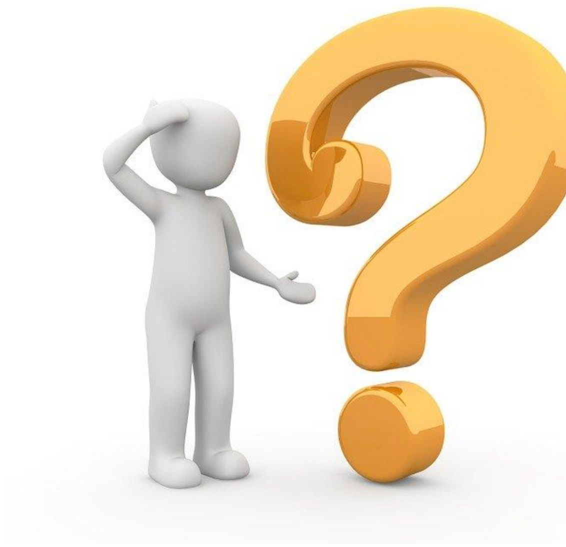

Answer 7

Correct answers: a, c, d

b is wrong: Lean fish does not contain significant amounts of vitamin D; fatty fish does.

Sources of vitamin D

Vitamin D is a fat-soluble hormone (referred to as a vitamin for historical reasons).
90% of vitamin D is produced in the skin with the help of the ultraviolet portion of sunlight.
A smaller amount (10%) comes from food: fatty fish (salmon, mackerel, etc.), eggs, mushrooms, dairy products.

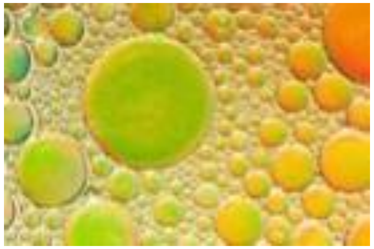

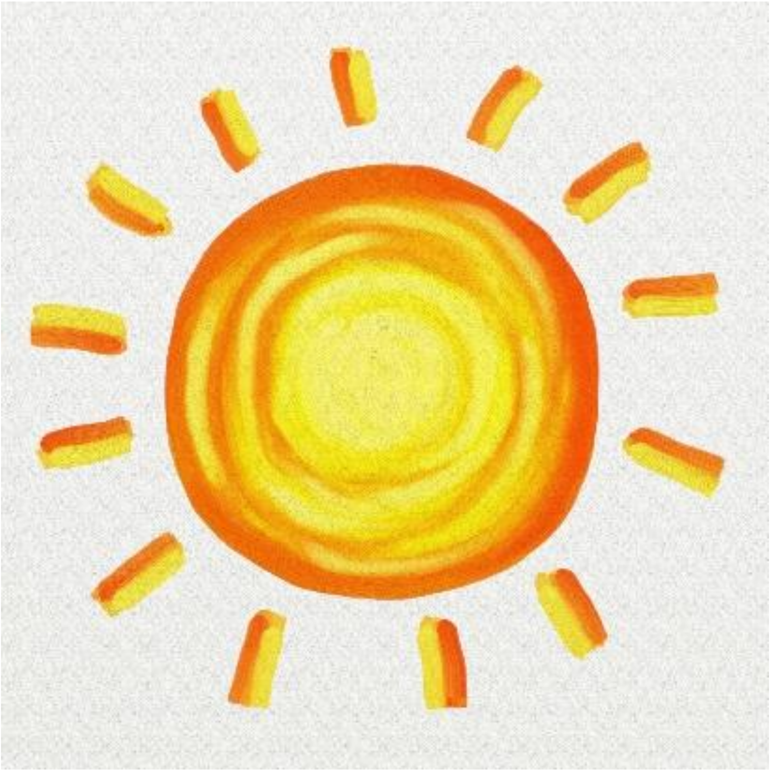

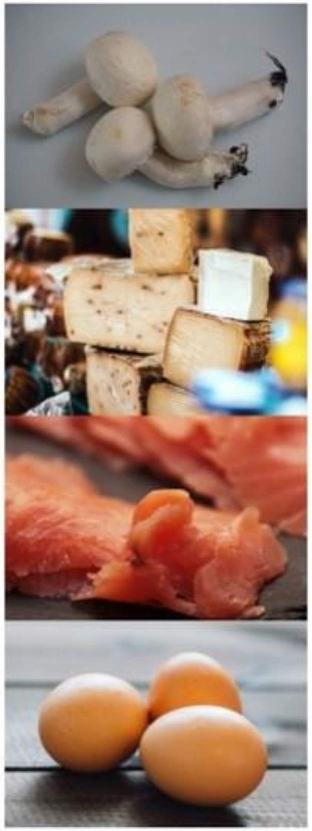

Algorithm: Vitamin D in the Inpatient Setting

MEASUREMENT

Disorder in calcium-phosphate metabolism or all three of the following criteria are met:

1) **Situations that can be improved by optimizing vitamin D levels**:
   - ✓ Osteoporosis, osteomalacia, rickets
   - ✓ Fractures with suspicion of osteoprosis (i.e., fracture without trauma or low trauma fractures)
   - ✓ Long-term oral corticosteroid use
   - ✓ Hyperparathyroidism
2) **No existing vitamin D supplementation**
3) **No previous normal vitamin D measurement**

→ 25-OH vitamin-D-measurement (nmol/l)

No disturbance in calcium-phosphate metabolism or ≥1 of the 3 criteria NOT met

→ No measurement

TREAMENT

25-OH vitamin D	Treatment
<25 nmol/l	6’000 IU/d for 6w, then 800 IU/d
25-49 nmol/l	800-1000 IU/d
≥ 50 nmol/l	Do not start supplementation<br>Continue existing maintenance dose of 400-800IU/d*
No measurement	Do not start supplementation<br>Continue existing maintenance dose of 400-800IU/d*

* As it is not wrong and the diagnosis list at hospital might be incomplete

Quiz 8

You are treating a patient who takes her husband's vitamin D drops daily because she read that vitamin D protects against viral infections. Her vitamin D level was 130 nmol/L. What do you explain to the patient?

a. The normal range is between 50-125 nmol/l.

b. The normal range is between 100-125 nmol/l.

c. The risk of harmful side effects increases above 125 nmol/l.

d. There is no evidence that taking vitamin D protects against viral infections.

e. The patient should continue taking vitamin D.

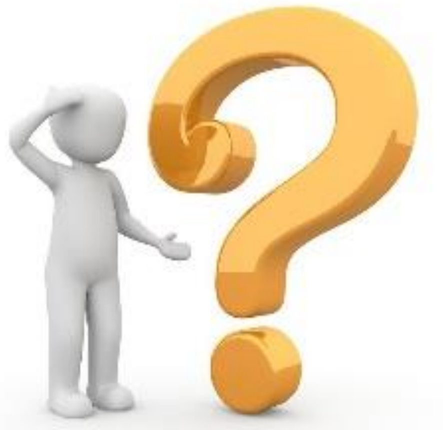

Answer 8

Correct answers: a, c, d

The normal range is 50-125 nmol/l, and supplementation is not recommended when levels exceed this range.

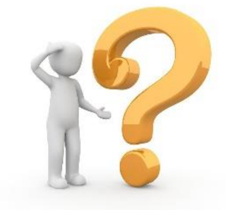

Vitamin-D-deficiency and -insufficiency

Vitamin D levels are categorized into 4 groups:

•Vitamin D **deficiency** is present when the 25-OH vitamin D level is <25 nmol/l.

•Vitamin D **insufficiency** is present when the 25-OH vitamin D level is 25-49 nmol/l.

•**Adequate** vitamin D supply is present when the 25-OH vitamin D level is 50-125 nmol/l.

•There is an increased risk of **side effects** from vitamin D when the 25-OH vitamin D level is ≥125 nmol/l.

Side effects of supplementation

Vitamin D supplementation at standard doses does **not** lead to:

- serious side effects
- nephrolithiasis
- hypercalcemia

Vitamin D intoxication only occurs with supplementation at excessively high doses. Patients present with symptoms of hypercalcemia.

Algorithm: Vitamin D in the Inpatient Setting

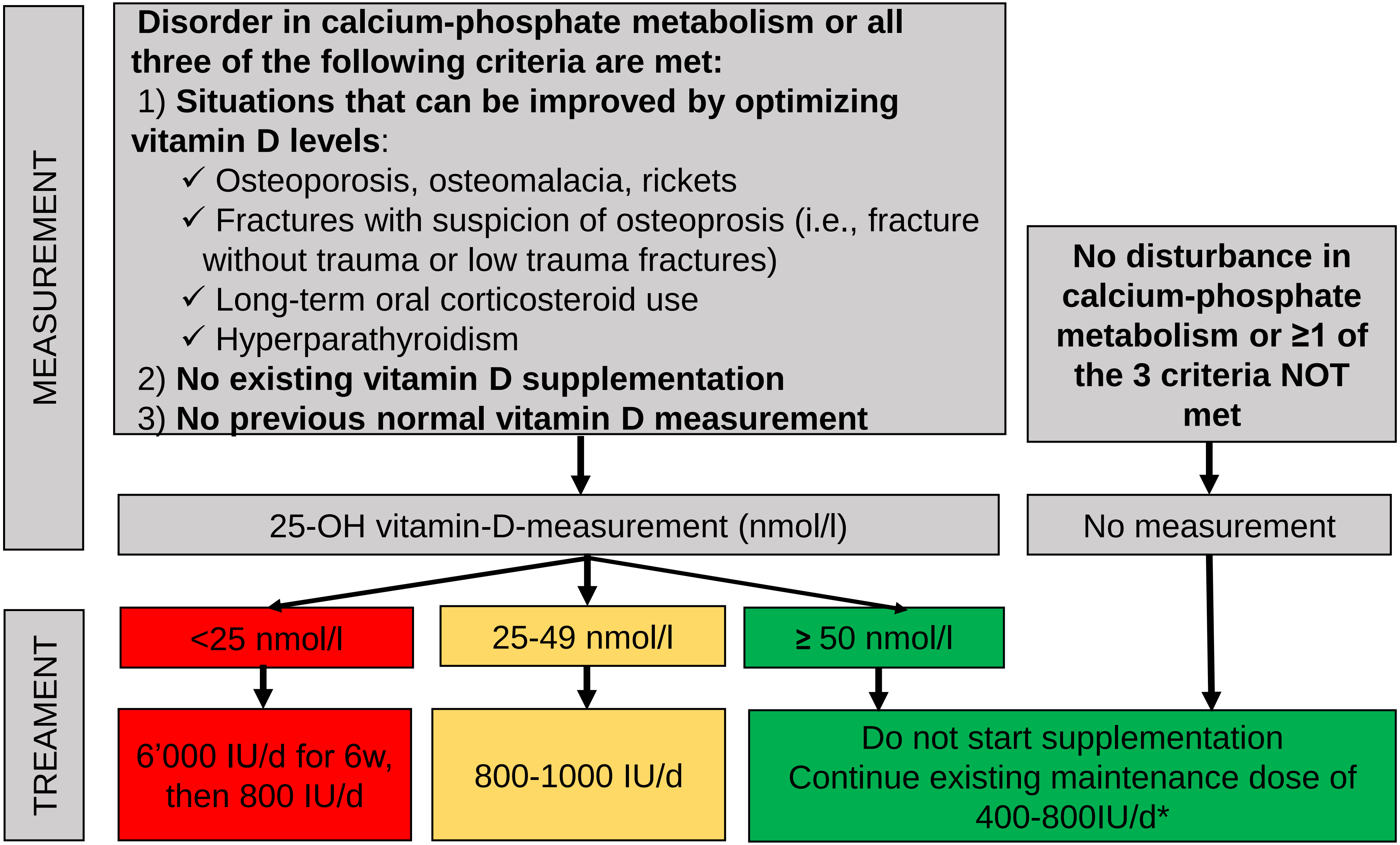

* As it is not wrong and the diagnosis list at hospital might be incomplete

Quiz 9

You treat a patient with secondary hyperparathyroidism and vitamin D deficiency. He does not respond to correctly dosed 25-OH vitamin D supplementation. What do you do?

a. Increase the dosage of supplementation.

b. Rule out malabsorption.

c. Switch from 25-OH vitamin D to 1,25-OH vitamin D (calcitriol).

d. Switch from oral to intramuscular administration.

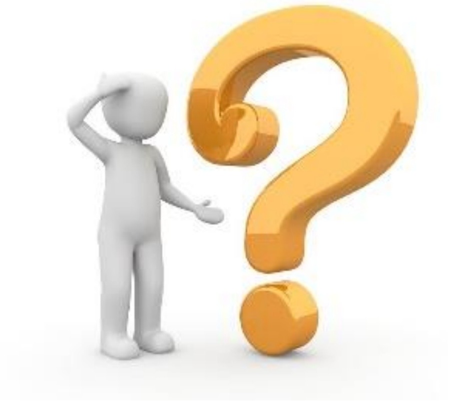

Answer 9

Correct answer: c

a: If there is no response to properly dosed 25-OH vitamin D supplementation and kidney disease is present, supplementation should be switched to 1,25-OH vitamin D.
b: Given the medical history, malabsorption is an unlikely cause of the lack of response.
D: This would be correct if the cause of the lack of response to vitamin D supplementation were malabsorption.

Which form of Vitamin D should be measured?

25-OH Vitamin D should be measured, even in cases of hyperparathyroidism of renal origin.

If the vitamin D level is <50 nmol/l in hyperparathyroidism of renal origin, supplementation with 25-OH vitamin D should be initiated.

If hyperparathyroidism persists despite an adequate 25-OH vitamin D level (50-125 nmol/l), the 1-OH hydroxylation process is not functioning properly.

In such cases, substitution with 1,25-OH vitamin D (Calcitriol) should be started without prior measurement of 1,25-OH vitamin D.

Algorithm: Vitamin D in the Inpatient Setting

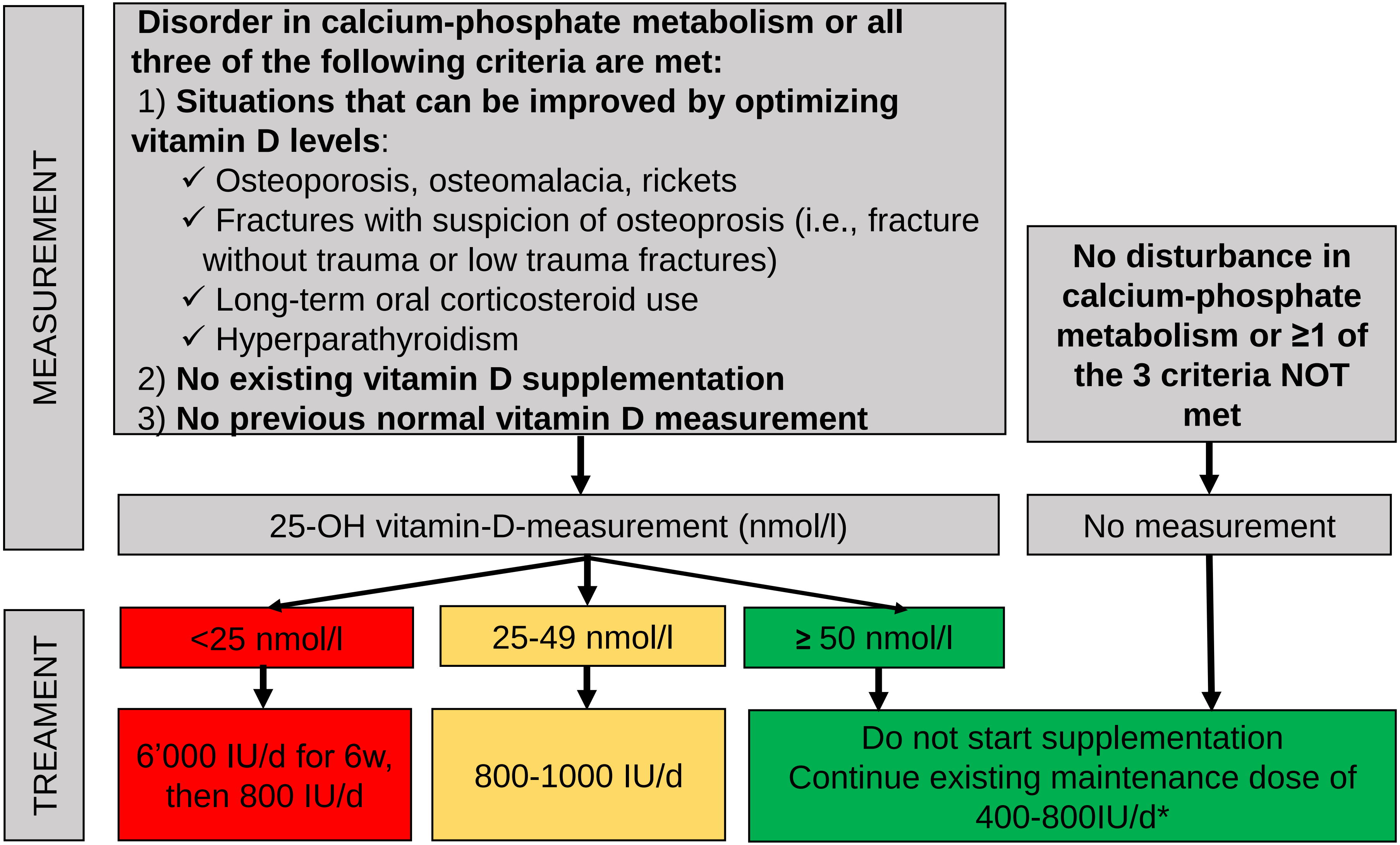

* As it is not wrong and the diagnosis list at hospital might be incomplete

Quiz 10

What are the indications for vitamin D supplementation?

a. Support of the immune system

b. Cancer prevention

c. Dementia prevention

d. Treatment of osteoporosis

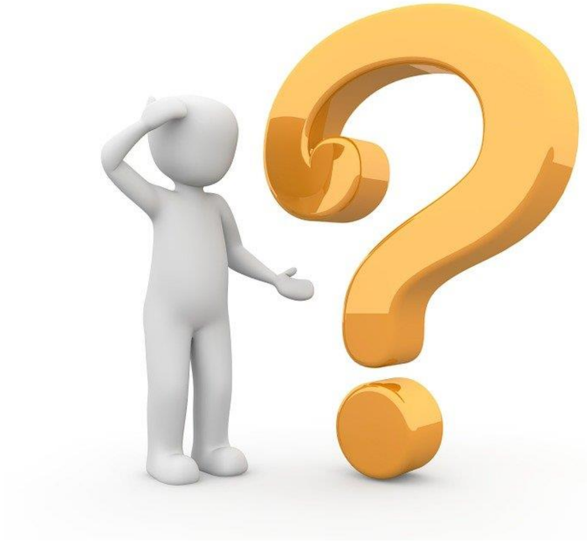

Quiz 10

Correct answer: d

There is no evidence that vitamin D supplementation supports the immune system or prevents cancer or dementia.

Benefits of supplementation

There is no benefit of vitamin D supplementation on the following outcomes in the general population:

- mortality
- physical performance
- prevention of fractures, diabetes mellitus, cancer, falls, depression, infections, cardiovascular events

Indications for Supplementation

50% of the general population in Switzerland has a vitamin D deficiency *(Benhamou et al. SMW 2021).*

However, there is **no evidence of the benefit** of supplementation in the general population

Supplementation should only be undertaken **if both of the following criteria** are met :

1) 25-OH Vitamin-D-level <50 nmol/l
2) A situation that can be improved by supplementation:
   - ✓ Osteoporosis
   - ✓ Osteomalacia
   - ✓ Rickets
   - ✓ Fractures suspected of osteoporosis (fractures without trauma or low-trauma fractures)
   - ✓ Long-term oral corticosteroid use (osteoporosis prevention)
   - ✓ Hyperparathyroidism (primary (due to more severe symptoms when vitamin D is low) and secondary)

Algorithm: Vitamin D in the Inpatient Setting

MEASUREMENT

Disorder in calcium-phosphate metabolism or all three of the following criteria are met:

1) **Situations that can be improved by optimizing vitamin D levels**:
   - ✓ Osteoporosis, osteomalacia, rickets
   - ✓ Fractures with suspicion of osteoprosis (i.e., fracture without trauma or low trauma fractures)
   - ✓ Long-term oral corticosteroid use
   - ✓ Hyperparathyroidism
2) **No existing vitamin D supplementation**
3) **No previous normal vitamin D measurement**

→ 25-OH vitamin-D-measurement (nmol/l)

No disturbance in calcium-phosphate metabolism or ≥1 of the 3 criteria NOT met

→ No measurement

TREAMENT

- <25 nmol/l → 6’000 IU/d for 6w, then 800 IU/d
- 25-49 nmol/l → 800-1000 IU/d
- ≥ 50 nmol/l → Do not start supplementation Continue existing maintenance dose of 400-800IU/d*
- No measurement → Do not start supplementation Continue existing maintenance dose of 400-800IU/d*

* As it is not wrong and the diagnosis list at hospital might be incomplete

Quiz 11

How do you supplement vitamin D in a patient with a vitamin D level of 20 nmol/L and osteoporosis?

a. 800-1000 IU/d

b. 1500 IU/d

c. 6000 IU/d for 6 weeks, then 800-1000 IU/d

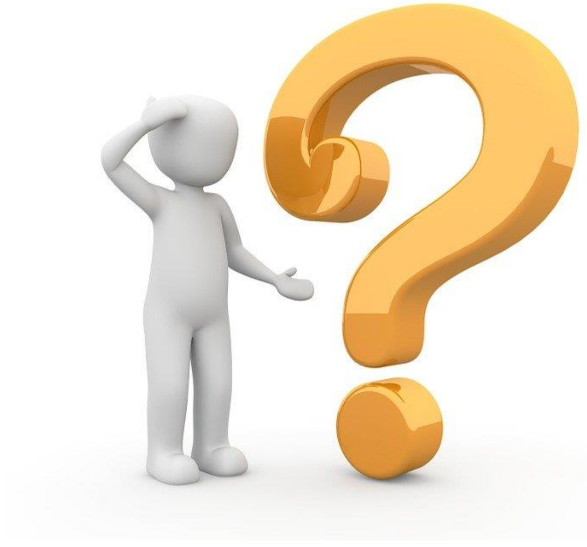

Answer 11

Correct answer: c

a: this is the dose for vitamin D insufficiency, not for vitamin D deficiency.

b: this dose is too low at the beginning of supplementation for vitamin D deficiency.

Supplementation Schemes

Supplementation with 100 IU of vitamin D leads to an increase in levels by 1.75-2.50 nmol/l.

There are two supplementation schemes, depending on vitamin D levels:

- for vitamin D **deficiency** (<25 nmol/l):
    - 100,000 IU every 2 weeks or 6000 IU/d orally for 6 weeks, followed by 800-1000 IU/d
- for vitamin D **insufficiency** (25-50 nmol/l):
    - 800-1000 IU/d

Algorithm: Vitamin D in the Inpatient Setting

MEASUREMENT

Disorder in calcium-phosphate metabolism or all three of the following criteria are met:

1) **Situations that can be improved by optimizing vitamin D levels**:
   - ✓ Osteoporosis, osteomalacia, rickets
   - ✓ Fractures with suspicion of osteoprosis (i.e., fracture without trauma or low trauma fractures)
   - ✓ Long-term oral corticosteroid use
   - ✓ Hyperparathyroidism
2) **No existing vitamin D supplementation**
3) **No previous normal vitamin D measurement**

→ 25-OH vitamin-D-measurement (nmol/l)

No disturbance in calcium-phosphate metabolism or ≥1 of the 3 criteria NOT met

→ No measurement

TREAMENT

- <25 nmol/l → 6’000 IU/d for 6w, then 800 IU/d
- 25-49 nmol/l → 800-1000 IU/d
- ≥ 50 nmol/l → Do not start supplementation
  Continue existing maintenance dose of 400-800IU/d*
- No measurement → Do not start supplementation
  Continue existing maintenance dose of 400-800IU/d*

* As it is not wrong and the diagnosis list at hospital might be incomplete

Quiz 12

How is active vitamin D produced?

a. 90% of vitamin D is produced in the skin with the help of UV rays from the sun.

b. The first hydroxylation occurs in the liver, and the second in the kidneys.

c. The first hydroxylation occurs in the kidneys, followed by a second in the liver.

d. Half of the vitamin D storage comes from food.

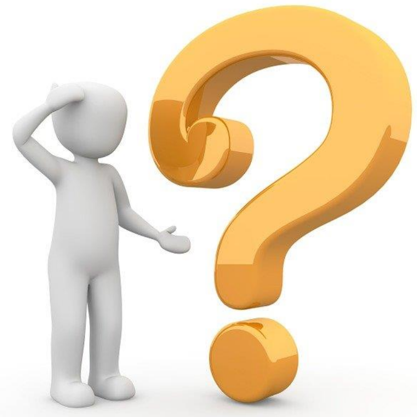

Answer 12

Correct answers: a and b

c: the first hydroxylation occurs in the liver, the second one in the kidney.

c: only 10% comes from nutrition. 90% of vitamin D are produced in the skin with the ultraviolets of the sun.

What is Vitamin-D?

Vitamin D is a fat-soluble hormone (referred to as a vitamin for historical reasons).
90% of vitamin D is produced in the skin with the help of the ultraviolet portion of sunlight.
A smaller amount (10%) comes from food: fatty fish (salmon, mackerel, etc.), eggs, mushrooms, dairy products.

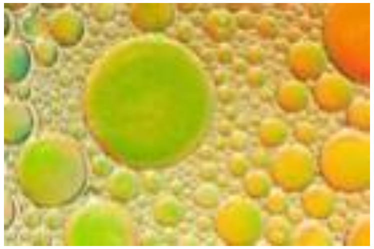

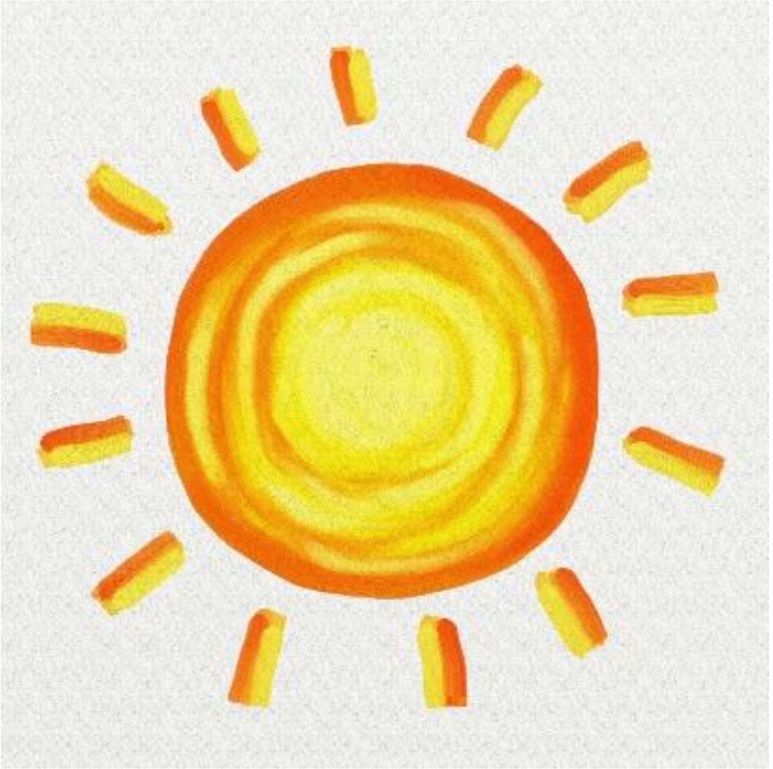

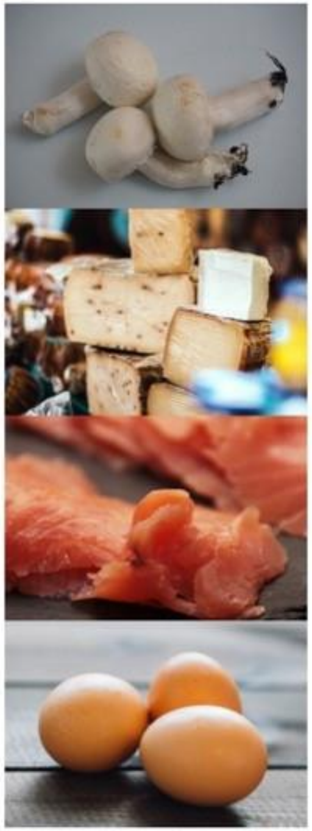

Activation of Vitamin D

Vitamin D (=vitamin D_3) is inactive

It requires 2 hydroxylations to become active.

The first hydroxylation occurs in the liver, producing 25-OH vitamin D.

The second hydroxylation occurs in the kidney, producing 1,25-OH vitamin D.

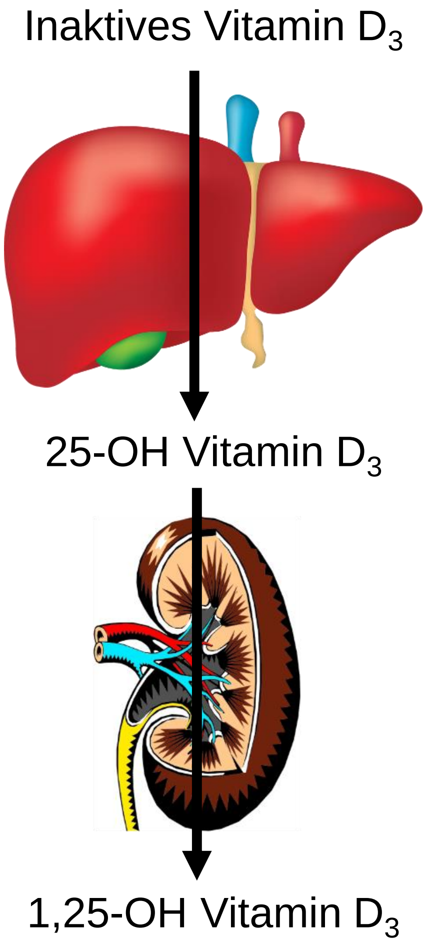

Algorithm: Vitamin D in the Inpatient Setting

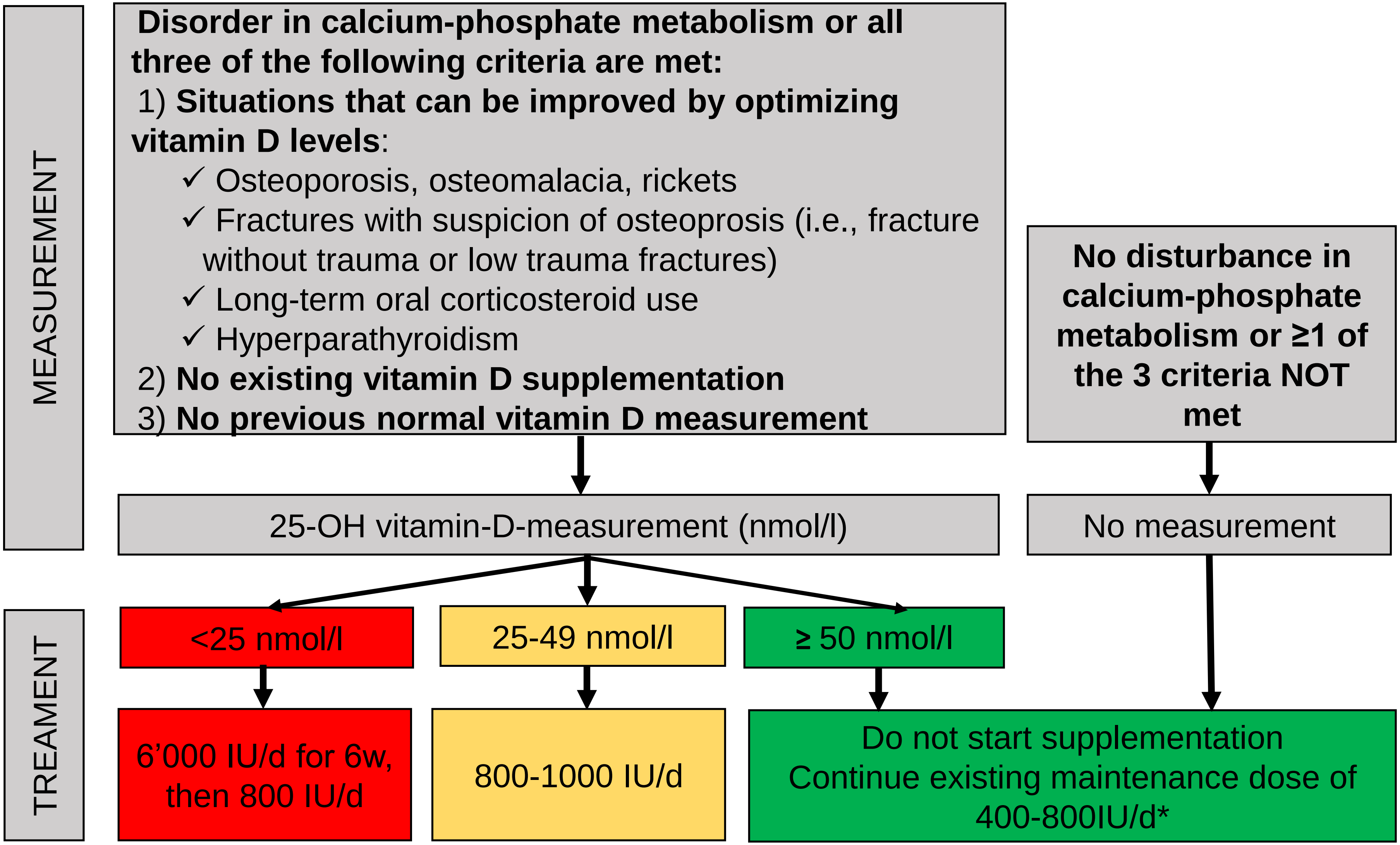

* As it is not wrong and the diagnosis list at hospital might be incomplete
